# Efficacy of Targeted Radionuclide Therapy Using [^131^I]ICF01012 in 3D Pigmented BRAF- and NRAS-Mutant Melanoma Models and In Vivo NRAS-Mutant Melanoma

**DOI:** 10.3390/cancers13061421

**Published:** 2021-03-20

**Authors:** Hussein Akil, Mercedes Quintana, Jérémy H. Raymond, Tommy Billoux, Valentin Benboubker, Sophie Besse, Philippe Auzeloux, Véronique Delmas, Valérie Petit, Lionel Larue, Michel D’Incan, Françoise Degoul, Jacques Rouanet

**Affiliations:** 1 INSERM U1240, University of Clermont Auvergne, 58 rue Montalembert, 63000 Clermont-Ferrand, France; hussein.akil@unilim.fr (H.A.); mercedes.quintana@inserm.fr (M.Q.); vbenboubker@chu-clermontferrand.fr (V.B.); sophie.besse@inserm.fr (S.B.); philippe.auzeloux@inserm.fr (P.A.); mdincan@chu-clermontferrand.fr (M.D.); francoise.degoul@inserm.fr (F.D.); 2 CNRS 7276, INSERM U1262, 2 rue du Pr Descottes, 87025 Limoges, France; 3 INSERM U1021, Normal and Pathological Development of Melanocytes, Institut Curie, PSL Research University, Campus Universitaire, 91898 Orsay, France; jeremy.raymond@curie.fr (J.H.R.); veronique.delmas@curie.fr (V.D.); valerie.petit@curie.fr (V.P.); lionel.larue@curie.fr (L.L.); 4 Campus Universitaire, University Paris-Sud, University Paris-Saclay, CNRS UMR3347, 91898 Orsay, France; 5 Equipes Labellisées-Ligue Contre le Cancer, Campus Universitaire, 91898 Orsay, France; 6 Cirmen, Centre Jean Perrin, 58 rue Montalembert, 63000 Clermont-Ferrand, France; Tommy.BILLOUX@clermont.unicancer.fr; 7 Department of Dermatology and Oncodermatology, CHU Estaing, 1 Place Aubrac, 63000 Clermont-Ferrand, France; 8 CNRS 6293 INSERM U1103, University of Clermont Auvergne, 28, Place Henri Dunant, 63000 Clermont-Ferrand, France

**Keywords:** targeted radionuclide therapy, MEK inhibitors, BRAF mutation, NRAS mutation, melanoma spheroid model

## Abstract

**Simple Summary:**

Targeted radionuclide therapy (TRT) aims to selectively deliver radioactive molecules to tumor cells. For this purpose, we deliver iodine-131 ([^131^I]) to melanoma cells by using our laboratory-developed melanin specific radiotracer, the ICF01012. Approximately 50% and 20%–30% of human melanomas have activating mutation in BRAF or NRAS genes, respectively. These mutations lead to a constitutive activation of the MAPK/ERK pathway, which is known to be involved in tumor cells’ radioresistance. In this work, we showed using 3D in vitro tumor models, an additive efficiency of combining [^131^I]ICF01012-TRT and MAPK/ERK inhibitors in BRAF- and NRAS-mutant melanoma cells. In mice bearing NRAS^Q61K^-mutated melanoma, TRT induced an impressive decrease in tumor growth, as well as a highly extended survival. Additionally, we showed that TRT reduces the metastatic capacity of melanoma, especially through lymph-node dissemination. These results are therefore of great interest, especially for patients with NRAS-mutant metastatic melanoma who currently lack specific efficient therapies.

**Abstract:**

Purpose: To assess the efficiency of targeted radionuclide therapy (TRT), alone or in combination with MEK inhibitors (MEKi), in melanomas harboring constitutive MAPK/ERK activation responsible for tumor radioresistance. Methods: For TRT, we used a melanin radiotracer ([^131^I]ICF01012) currently in phase 1 clinical trial (NCT03784625). TRT alone or combined with MEKi was evaluated in three-dimensional melanoma spheroid models of human BRAF^V600E^ SK-MEL-3, murine NRAS^Q61K^ 1007, and WT B16F10 melanomas. TRT in vivo biodistribution, dosimetry, efficiency, and molecular mechanisms were studied using the C57BL/6J-NRAS^Q61K^ 1007 syngeneic model. Results: TRT cooperated with MEKi to increase apoptosis in both BRAF- and NRAS-mutant spheroids. NRAS^Q61K^ spheroids were highly radiosensitive towards [^131^I]ICF01012-TRT. In mice bearing NRAS^Q61K^ 1007 melanoma, [^131^I]ICF01012 induced a significant extended survival (92 vs. 44 days, *p* < 0.0001), associated with a 93-Gy tumor deposit, and reduced lymph-node metastases. Comparative transcriptomic analyses confirmed a decrease in mitosis, proliferation, and metastasis signatures in TRT-treated vs. control tumors and suggest that TRT acts through an increase in oxidation and inflammation and P53 activation. Conclusion: Our data suggest that [^131^I]ICF01012-TRT and MEKi combination could be of benefit for advanced pigmented BRAF-mutant melanoma care and that [^131^I]ICF01012 alone could constitute a new potential NRAS-mutant melanoma treatment.

## 1. Introduction

Cutaneous malignant melanoma is one of the most lethal forms of skin cancer. It develops from epidermal melanocytes responsible for melanin synthesis [1]. These pigments protect melanocytes and neighboring keratinocytes from deoxyribonucleic acid (DNA) double-strand breaks by forming a shield that absorbs reactive oxygen species (ROS) induced by ultraviolet (UV) radiation. The main signaling pathway involved in melanomagenesis is that of mitogen-activated protein kinases (MAPK), along with the associated *Rat sarcoma* GTPase (RAS)/*rapidly accelerated fibrosarcoma* protein(RAF)/mitogen-activated extracellular signal-regulated kinase kinase (MEK)/*extracellular signal-related kinase* (ERK proteins) [1,2]. Somatic mutations in genes encoding protein actors of this phosphorylation cascade constitutively activate the MAPK pathway: mutations in *BRAF*, neurofibromin 1 (*NF1*)*, NRAS,* and *c-KIT* are the most frequent, representing 50, 20, 20, and 2% of melanomas, respectively [3]. Most (70 to 90%) mutations in *BRAF* lead to the substitution of valine by glutamic acid at position 600 in BRAF (V600E). Mutations in NRAS gene occur in 20–30% of melanomas, at codon 61 for ≈90% of the cases, resulting in a change in amino acid Q61K (45%) and Q61R (35%) while Q61L and Q61H modifications were lower (20%). Other genetic alterations in NRAS gene G12R/D/A/V occurred at a low rate (≈10%) [3,4].

Based on these molecular characteristics of the melanoma, specific BRAF (V600E) ATP-competitive inhibitors, such as vemurafenib [5] or dabrafenib [6] have been developed and have shown remarkable clinical efficacy in BRAF(V600E) melanoma tumors. Despite the initial positive effects on melanoma progression, these selective inhibitors often lead to the development of rapid neo/acquired resistance, resulting in either reactivation of the MAPK signaling pathway or activation of PI3K signaling pathway [2,7,8], usually within 6 to 8 months of treatment [9]. Several mechanisms involved in resistance to BRAF inhibitors have been identified and a number of strategies combining them with MEK inhibitors have been tested in multiple phase 3 trials. For example, the COMBI-d and COMBI-v trials studied the efficiency of combining dabrafenib with a MEK inhibitor, trametinib, compared to dabrafenib or vemurafenib alone [9]. Five-year pooled analyses [10] showed that this combination leads to a progression free survival (PFS) rate of 19%, with a median PFS of 11.1 months, and an overall survival (OS) rate of 34%, with median OS of 25.9 months. For patients receiving only dabrafenib or vemurafenib, the 5-year PFS rate was 13% and 9%, respectively; the 5-year OS rate was 27% and 23%, respectively, confirming the long-term interest of combining BRAF and MEK inhibitors. The phase 3 coBRIM [11] trial compared vemurafenib and cobimetinib vs. placebo and vemurafenib and obtained a median PFS of 12.6 months for the combination vs. 7.2 months for vemurafenib alone. The overall response rate (ORR) was 70% vs. 50% and median OS was 22.5 vs. 17.4 months. The most recent trial [12,13] (COLUMBUS) assessed the combination of binimetinib (MEK inhibitor) with encorafenib (BRAF inhibitor). This study showed the best efficiency for a BRAF/MEK combination versus vemurafenib alone and a very low number of adverse events, such as photosensitivity or fever. The combination showed an ORR of 64% and median PFS of 14.9 vs. 7.3 and 9.6 months for vemurafenib and encorafenib, respectively [13]. OS was 33.6 months for the combination vs. 16.9 months for vemurafenib (*p* ≤ 0.0001) [13]. Moreover, the number of secondary cutaneous cancers was lower with combination therapy in both trials. These significant clinical benefits have made combined BRAF and MEK inhibitors the new standard for advanced and metastatic V600E BRAF-mutated melanoma treatment. There are no clinically approved inhibitors for NRAS mutant melanomas. Initial clinical trials have tested farnesyl transferase inhibitors (FTIs), which block the lipid post-translational modification of RAS required for its activity. One such FTI (tipifarnib) was clinically tested but showed no significant clinical benefit [14]. The NRAS inhibitor salirasib, which modifies RAS-GTP binding to cell membranes by competition, has not been assessed in a clinical trial for melanoma [15]. MEK inhibitors have been tested in preclinical and clinical studies with promising results. However, the phase 3 NEMO trial, which compared binimetinib to dacarbazine, failed to show a benefit on OS (11 vs. 10 months, hazard ratio (HR) 1.00 (95% CI (0.75–1.33)); one-sided *p* = 0.50) although there was an extension of PFS (2.8 vs. 1.5 months, HR 0.62 (95% CI [0.47–0.80]); one-sided *p* < 0.001) [16]. Recruiting has been halted due to poor results. Thus, immune checkpoint inhibitors [9] are currently the sole treatment available for patients with NRAS melanoma. 

Targeted radionuclide therapy (TRT) consists in delivering radiopharmaceuticals to tumor cells by targeting specific characteristics **[17]**. [^131^I]ICF01012 is a melanin-targeting compound that has already shown its efficiency by reducing tumor growth and enhancing survival in various syngeneic and xenograft pigmented melanoma models with wild-type or mutant BRAF [18,19,20,21], whereas injection of [^131^I] alone or of non-labeled ICF01012 in mice bearing B16 melanomas did not modify mice survival and tumor growth [21]. In addition, biodistribution studies and secondary ion mass spectrometry (SIMS) technique have confirmed a specific accumulation of [^131^I]ICF01012 in pigmented tissues/cells and in acidic organelles [18,19,20,21,22,23]. [^131^I]ICF01012 can also reduce melanoma metastases, mainly by modifying pseudo-epithelial-mesenchymal transition mechanisms, in vitro and in vivo, in murine and human melanoma cell lines [24]. We have also shown that TRT using [^131^I]ICF01012 induces first a decrease and then an insignificant increase in ERK phosphorylation [18], suggesting a mechanism of radioresistance through activation of the MAPK pathway.

The association of targeted therapies and radiation has already shown positive results in preclinical [25,26] and clinical studies [27]. Indeed, BRAF and MEK inhibitors have been shown to increase melanoma-cell radiosensitivity [25]. Hecht et al. have confirmed that combined radiotherapy and administration of BRAF inhibitors is tolerable, with an acceptable increase in toxicity [27]. We developed a three-dimensional (3D) melanoma spheroid approach for in vitro studies of TRT to mimic tumor architecture and better model the consequences of irradiation [24]. Here, we investigated the effect of combining MEK inhibitors with [^131^I]ICF01012-TRT in an in vitro 3D spheroid model. We further studied the efficiency of [^131^I]ICF01012-TRT in vivo using the syngenic NRAS^Q61K^ 1007-mutant melanoma allograft model corresponding to the human NRAS^Q61K^ mutation [28].

## 2. Materials and Methods

### 2.1. Cell Lines, Culture Conditions and Spheroid Collection

Human SK-MEL-3 and murine B16F10 melanoma cell lines were purchased from the American Type Culture Collection (ATCC). SK-MEL-3 cells were maintained as monolayer using culture medium consisting of McCOY’S 5A medium (Invitrogen, Cergy Pontoise, France) supplemented with 15% fetal calf serum (FCS) (Eurobio, Les Ulis, France), and 4 µg µL^−1^ gentamycin (Invitrogen) at 37 °C in a humidified incubator containing 5% CO_2_. B16F10 cells were maintained as monolayer using culture medium consisting of DMEM-Glutamax medium (Invitrogen) supplemented with 10% FCS (Eurobio), and 4 µg µL^−1^ gentamycin (Invitrogen) at 37 °C in a humidified incubator containing 5% CO_2_. The NRAS^Q61K^ 1007 (also named NRAS 1007) murine cell line was cultured as previously described [28]. Melanoma spheroids were generated as previously described [24].

Spheroids were harvested between 1 and 72 h post-[^131^I]ICF01012 removal, frozen in N_2_, and stored at −80 °C during the radioactive decay (80 days; 10 times the half-life of the iodine-131 isotope) for Western blot analysis or fixed in 70% ethanol for cell-cycle studies.

### 2.2. [^131^I]ICF01012 TRT and MEK Inhibitor Treatment of Spheroids

[^131^I] iodine was purchased from Perkin Elmer (Waltham, MA, USA) or Izotop (Budapest, Hungary). Radiolabeling of ICF01012 was performed as previously described [23]. For the [^131^I]ICF01012 alone experiments, each spheroid was irradiated at day 6 of culture with 37 kBq of [^131^I]ICF01012/100 µL of cell culture medium (without FCS). The addition of medium alone was used as a control. After 1 h of incubation, the irradiation medium was removed and replaced by complete medium supplemented with 0.5% methylcellulose. For the [^131^I]ICF01012 and MEK inhibitors (MEKi) combinations, each spheroid was pretreated for 2 h with cobimetinib (GDC-0973) (Selleckchem (Houston, TX, USA), 100 nM, SK-MEL-3 spheroids), GDC-0623 (Selleckchem, 50 nM, B16F10, and NRAS 1007 spheroids), or dimethyl sulfoxide (DMSO) (control and [^131^I]ICF01012 alone groups) on day 6 of culture. Then, each spheroid was irradiated according to the above-mentioned protocol with cell culture medium containing the corresponding MEKi (MEKi alone or TRT + MEKi groups) or DMSO (control DMSO or TRT alone groups). Spheroids were then incubated from 1 to 72 h.

### 2.3. Colony Formation Assay

Twenty-four hours after irradiation in combination with MEKi (as described above for the combination [^131^I]ICF01012 and MEKi), 60 spheroids/condition were collected and centrifuged (50 g, 5 min). Supernatants were removed and spheroids were dissociated with 500 µL collagenase IV (0.2%; Sigma Aldrich, Saint-Quentin Fallavier, France) during 30 min at 37 °C. Cells were re-suspended, counted, and seeded in 6 well plates with a 2 mL final volume of complete culture medium containing the corresponding MEKi (TRT + MEKi and MEKi groups) or DMSO (TRT and control groups). SK-MEL3 cells were seeded at 7200 cells/well, NRAS 1007 at 5000 cells/well, and B16F10 at 400 cells/well. After 3 days of incubation, culture medium was removed and replaced by complete culture medium without DMSO or MEKi. After 18 days of incubation for SK-MEL3, 15 days for NRAS 1007, and 8 days for B16F10 cells, medium was removed and colonies were rinsed once with PBS. Colonies were then fixed by methanol absolute during 3 min then revealed with 0.5% crystal violet aqueous solution. The counting of colonies was realized using the ImageJ software. Plating efficiency (PE) and survival fraction (SF) were determined as described [29]. 

### 2.4. Apoptosis Assay

Apoptosis was measured by the detection of cytoplasmic soluble nucleosomes using a colorimetric assay, Cell Death Detection ELISAPLUS (Sigma Aldrich) according to the manufacturer’s instructions. Absorbance was measured at 405–490 nm dual wavelengths.

### 2.5. Cell Cycle Analysis

Spheroids were dissociated with collagenase IV (0.2%; Sigma Aldrich) during 30 min at 37 °C. Cells were then fixed in 70% ethanol and stored at −20 °C. After complete radioactive decay, cells were washed twice with PBS then 50µL of Ribonuclease A (100 µg mL^−1^; Sigma-Aldrich) and 450 µL of propidium iodide (50 µg mL^−1^; Sigma-Aldrich) were added. After 15 min of incubation in the dark at room temperature, cell cycle was analyzed using a flow cytometer (BD Accuri C6 Plus, BD Biosciences, Le Pont de Claix, France).

### 2.6. Western Blotting

Western blot analysis was carried out as previously described [30]. The following primary antibodies (Abs) were used: anti-phospho-H2A.X (S139) (1/2000), anti-H2A.X (1/1000), anti-phospho-ERK1/2 (Thr202/Tyr204) (1/2000), anti-ERK1/2 (1/2000), anti-phospho-MEK1/2 (Ser217/221) (1/2000), and anti-MEK1/2 (1/2000) from Cell Signaling Technology (Danvers, MA, USA), and anti-PARP-1 (1/200; Santa Cruz Biotechnology, (Dallas, TX, USA)) and anti-Actin (1/10,000; Sigma-Aldrich). The following secondary Abs were used: anti-rabbit-HRP (1/5000) and anti-mouse-HRP (1/5000 and 1/10,000 for actin) from SouthernBiotech (Nanterre, France). The Western blots were scanned using ChemiDoc imaging system (Bio-Rad, Marnes-la-Coquette, France). The densitometric analyses were performed using an *ImageJ* software (National Institutes of Health, Bethesda, MD, USA) (accessed on 11 November 2020).

### 2.7. Murine Models

This investigation conformed to the Guide for the Care and Use of Laboratory Animals published by the US National Institutes of Health (8th edition, 2011). All experiments were conducted in accordance with the relevant guidelines and regulations and approved by both the local ethics committee of Clermont-Ferrand (C2E2A n°002) and the French Ministry of Education and Research (approval n°12211-2017111613576925). NRAS 1007 melanoma cells (1 × 10^5^) in 100 µL of saline solution were injected subcutaneously in the right flank of five-week-old C57BL/6J male mice purchased from Charles River Laboratories (Ecully, France). Three different in vivo experiment using [^131^I]ICF01012 were realized: a biodistribution and dosimetry study using 3 animals per time (n = 15), a survival study (n = 28), and a mechanistic study (n = 15) including single-photon emission computed tomography (SPECT-CT) imaging, melanin quantification, lymph nodes, and transcriptomic analyses.

### 2.8. Biodistribution Study

Thirty-six days following tumor implantation, 15 mice received an intra-venous (i.v.) injection of 0.37 MBq [^131^I]ICF01012. Three mice were sacrificed per timepoint (1, 3, 6, 24, and 72 h) prior to removing and weighing the organs and tumors. Their radioactivity was analyzed using a γ-counter (Wizard 1480, Perkin Elmer) and the gamma counting data were corrected for physical decay and background. The percentage of injected activity in tumors was determined by the ratio of counted activity per organ divided by the organ weight (% IA/g).

### 2.9. Dosimetry

The dose was assessed from organ activity measured at each timepoint (1, 3, 6, 24, and 72 h, n = 3 per timepoint).

The percentage of injected activity curves $\%IA\left(t\right)=\frac{Activity\left(t\right)}{weight.Activity_{injected}}$ were calculated for each organ. Curves were adjusted using MatLab software (TheMathWorks, Natick, MA, USA): tumor: $y=a\left(1-exp^{-bt}\right)exp^{-ct}\;$; eyes: $y=a\left(1-exp^{-bt}\right)exp^{-\lambda_{physique}t}$ with $\lambda_{physique}=\frac{\ln2}{T_{physique\left(h\right)}}\;$; other organs (bi-exponential): $y=a.exp^{-bt}+c.exp^{-dt}$.

The MatLab adjustment parameters were: Method: NonlinearLeastSquare; Robust: Bisquare; Algorithm: Trust-Region. Cumulative activity confidence intervals were assessed by bootstrapping (adjustment with a 153-fit simulation and the leave-one-and-two-out method) and 95% CI, with the 2.5 and 97.5 percentiles. Mouse and human doses were calculated with MIRD21 formalism, using S factors from Perrot et al. [31] for mice and Olinda factors and organ weights for humans [32].

### 2.10. [^131^I]ICF01012 Treatment

C57BL/6J mice were injected intravenously with either 18.5 MBq/100 µL of [^131^I]ICF01012 (TRT groups) or 100 µL saline (control groups) 36 days after tumor implantation. Mice were sacrificed before the tumor volume reached 1500 mm^3^ for survival study or 10 days after TRT injection for mechanistic analyses (including SPECT-CT imaging, melanin quantification, lymph nodes and transcriptomic analyses). Mice were homogeneously randomized between control and treated groups according to mice weight and tumor size, seven days after cells injection (survival study: TRT: n = 14, control: n = 14; mechanistic study: TRT: n = 8, control: n = 10).

Body weight and tumor volume were measured three times a week until tumor volume reached 1000 mm^3^ and then, daily (For mice weight and tumor volume at start of experiment and endpoint for survival and mechanistical studies, see Supplementary Table S1). Potential toxicities were evaluated daily with a scoring grid, exploring general condition, behavior changes, and skin toxicities. Mice were sacrificed when tumor volume reached approximately 1500 mm^3^ or at experiment time.

Tumor volume was calculated from the measurement of two perpendicular diameters using a caliper according to the formula L × S2/2, where L and S are the largest and smallest diameters, respectively, expressed in millimeters.

The doubling time was calculated individually for each animal. Tumor growth has an exponential function (N = N0.e^at^), the doubling time (DT) is calculated using the formula DT = Ln2/a, where “N” represents the volume of the tumor, “N0” represents the volume of the tumor at time 0, “a” represents the slope of the exponential phase, and “t” the time (in days).

### 2.11. SPECT-CT Imaging

Multimodal SPECT-CT imaging was performed using a NanoScan SPECT/CT camera (Mediso Ltd., Budapest, Hungary) equipped with four detectors and multi pinhole collimation (APT62). Mice were placed in a Multicell Mouse L bed (Mediso Ltd.) with temperature control (37 °C). SPECT-CT imaging was performed on representative mice (n = 2 at 1 h and 6 h and n = 5 at 24 h, 72 h, and 168 h) of each group at different time points (i.e., 1 h, 6 h, 24 h, 72 h and 168 h) after the injections of [^131^I]ICF01012. Nucline software (Mediso Ltd.) was used for image acquisitions and reconstructions (Nucline 3.00.018). CT parameters were as follows: helical scan with 480 projections (300 ms per projection), 50 kV, 590 uA, pitch 1.0, binning 1:4, and field of view: max. SPECT images were acquired within the CT scan range, with a standard resolution. The time per projection was determined in accordance to the detected radioactivity (most frequently used: 30 s). SPECT image reconstruction was conducted using TeraTomo3D (Nucline v3.00.018) with high dynamic range. Regularization filters, reconstruction resolution and iterations were set to “medium”. Additional corrections were performed during reconstruction: Monte Carlo correction quality was set to “high”; attenuation: based on CT attenuation map and scatter corrections; activity decay correction: during acquisition time lapse.

### 2.12. Melanin Determination

For melanin assays, tumors were excised 10 days after [^131^I]ICF01012 irradiation, frozen in N2, and stored at −80°C for radioactive decay (80 days; 10 times the half-life of the iodine-131 isotope). Eumelanin and pheomelanin analyses were performed as previously described [33].

### 2.13. RT-qPCR Analyses

Inguinal and axillar lymph nodes (LNs) were collected 10 days after [^131^I]ICF01012 irradiation, frozen in N_2_, and stored at −80 °C. After complete radioactive decay, ribonucleic acid (RNA) was extracted (RNA Extraction Kit, Macherey Nagel) and the amounts measured by spectrophotometry (MultiskanGo, ThermoFisher, FisherScientific, Illkirch, France). Complementary DNA (cDNA) was synthesized from 250 ng RNA using the Thermoscript kit (ThermoFisher). Quantitative PCR (qPCR) reactions were performed in triplicate with Master Mix SybrGreen using an Applied BioSystems StepOne Plus device. Primers and annealing conditions are described in Supplementary Table S2. Results are expressed according to the ΔΔCT method after normalization against the glyceraldehyde 3-phosphate dehydrogenase (GAPDH) housekeeping gene.

### 2.14. Transcriptomic Analysis

According to Petit et al. [28], RNA from mouse melanomas (Control: n = 6, TRT: n = 6) was extracted using the miRNeasy Kit (Qiagen, Courtaboeuf, France, #217004). RNA integrity was assessed using an Agilent BioAnalyser 2100 (Agilent Technologies, Les Ulis, France), only RNA with a RNA integrity number (RIN) > 7 were kept for the analysis. This threshold led to the sequencing of 6 controls and 3 treated tumors. RNA concentrations were measured using a NanoDrop (NanoDrop Technologies, Wilmington, DA, USA). RNA sequencing libraries were prepared from 1 µg of total RNA using the Illumina TruSeq Stranded mRNA Library preparation kit that allows to perform a strand specific sequencing. A first step of polyA selection using magnetic beads is done to focus sequencing on polyadenylated transcripts. After fragmentation, cDNA synthesis was performed and resulting fragments were used for dA-tailing followed by ligation of TruSeq indexed adapters. PCR amplification was finally achieved to generate the final barecoded cDNA libraries (12 amplification cycles). The 9 libraries were equimolarly pooled and subjected to qPCR quantification using the KAPA library quantification kit (Roche). Sequencing was carried out on the NovaSeq 6000 instrument from Illumina based on a 2 × 100 cycles mode (paired-end reads, 100 bases) using an S1 flow cell in order to obtain around 35 million clusters (70 million raw paired-end reads) per sample. Reads were mapped to the mouse reference genome (gencode m13 version-GRCm38.p5) using STAR [34]. STAR was also used to determine the expression matrix. Expression matrix was first filtered by expression level to keep only sufficiently expressed genes using the edgeR package [35]. Principal component analysis (PCA) was performed using FactomineR [36]. 3D PCA animated gif was made with the help of the pca3d package (https://cran.r-project.org/web/packages/pca3d/index.html) (accessed on June 2020). The significance of the clustering was evaluated using PERMANNOVA. The deconvolution was conducted using the R-package mcp-counter [37]. To the gene set distributed with the package have been added two gene sets containing genes respectively specific of melanocytes (*Mitf*, *Pax3*, *Mc1r*, *Rxrg,* and *Tspan10*) and of keratinocytes (*Flg*, *Krt1*, *Dsc1*, *Sprr1b,* and *Krt14*) in the skin. Differential gene expression was performed with R following the limma-voom pipeline using the limma package [38]. edgeR and limma packages are both available from Bioconductor (http://www.bioconductor.org) (accessed on June 2020). The threshold for significantly differentially expressed genes was set as an absolute fold-change > two times the standard deviation of the fold-change and an adjusted *p*-value < 0.05. The volcano plot depicting the results was generated using the R package ggplot2 [39]. The interactive volcano plot was made with the plotly R package [40]. Gene-ontology and enriched pathway analysis were performed using Enrichr [41] on genes found overexpressed by the differential analysis either in the TRT-treated tumors or in the control tumors (also designed as CTRL-treated tumors. Gene-set enrichment analysis (GSEA) was performed using three collections from the Molecular Signatures Database (MSigDB v7.2): H (Hallmark), C2 (curated gene sets), and C5 (ontology gene sets), and a gene set describing the different sub-population in melanoma [42]. One thousand permutations gene set-based were made per analysis. The enrichment score (ES) reflects the degree to which a given gene set is represented in a ranked list of genes. Calculation of the ES is based on walking down a ranked list of genes and adjusting a running-sum statistic based on the presence of absence of a gene in the gene set. The magnitude of the increment represents the correlation of the gene with the phenotype. *p*-values were estimated by gene-based permutation. GSEA normalizes the enrichment score for each gene set to account for the variation in set sizes, yielding a normalized enrichment score (NES). Only gene sets with an NES > 2 and an FDR < 0.01 were considered. 

### 2.15. Statistical Analyses

Statistical analyses were performed with GraphPad (Addinsoft, New York, NY, USA) and StatView (Abacus concepts, Southfield, MI, USA) software using Log-rank for survival analyses, Student’s t tests for animal experiments, and two-way analysis of variance (ANOVA) associated with post-hoc Tukey’s multiple comparison test for in vitro experiments. A *p*-value < 0.05 was considered to represent statistically significant differences.

## 3. Results

### 3.1. MEKi Radio-Sensitizes BRAF- and NRAS-Mutant Melanoma Spheroids for [^131^I]ICF01012 by Increasing Apoptosis

**Figure 1:**
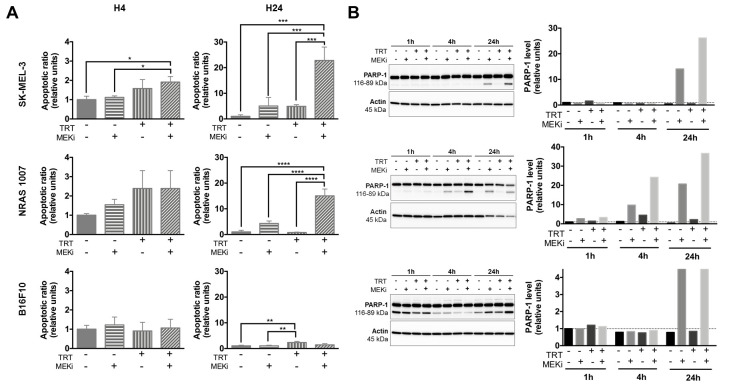
Apoptosis assays (n = 3) on melanoma spheroids after treatment with [^131^I]ICF01012-targeted radionuclide therapy (TRT), MEK inhibitors (MEKi), or both. Results are presented as mean +/− SD (**A**). (**A**) Apoptosis was assessed by quantification of soluble nucleosomes in cells using an ELISA cell death for SK-MEL-3, NRAS 1007, and B16F10. (**B**) Western blot analysis of cleaved PARP-1 in SK-MEL-3, NRAS 1007, and B16F10 spheroids treated with TRT, MEKi, or both. Actin was used as a protein-loading control. For Western blot, spheroids from 3 different experiments were pooled. For Western blot graphs, y-scales are not identical between cell lines. (* *p* < 0.05, ** *p* < 0.01, *** *p* < 0.001, **** *p* < 0.0001).

We first defined a 100-nM dose of cobimetinib to treat human melanoma SK-MEL-3 spheroids and a 50-nM dose of GDC-0623 for both the NRAS 1007 and B16F10 murine melanoma spheroids (Supplementary Figure S1). We further assessed the efficiency of combining TRT with MEKi using apoptosis assay. We showed that combining TRT with MEKi increased the apoptotic ratio of SK-MEL-3 spheroids by nearly 2-fold at 4 hours (h) post-irradiation and 22-fold at 24 h relative to either treatment alone: DMSO (*p =* 10^−4^), MEKi (*p* = 5 × 10^−4^), or TRT (*p* = 5 × 10^−4^) (Figure 1A). These results also showed a supra-additive mechanism. This effect was confirmed by Western blot analysis of cleaved poly (ADP-ribose) polymerase 1 (PARP-1) protein expression (Figure 1B). At 24 h post-irradiation, apoptotic ratio of NRAS 1007 spheroids (Figure 1A) treated with TRT + MEKi was nearly 15-fold higher than those treated with DMSO (*p <* 0.0001), MEKi alone (*p <* 10^−4^), or TRT alone (*p <* 10^−4^). Cleaved PARP-1 analysis also showed such radiosensitization, with increased levels of cleaved PARP-1 protein expression at 4 and 24 h post-irradiation (Figure 1B). These results highlight the high radiosensitivity of NRAS 1007 spheroids to TRT, which can be enhanced using MEKi. Consistent with our previous findings [18], a small percentage of B16F10 cells entered into apoptosis following TRT. As expected, MEKi did not induce significant apoptosis in these wild-type BRAF/NRAS spheroids (Figure 1A). These results were also confirmed by Western blot analysis, showing the absence of PARP-1 cleavage induction (Figure 1B).

These data suggest that, except for B16F10 spheroids, MEKi and TRT can cooperate to increase apoptosis in melanoma cells with a constitutively activated MAPK pathway (SK-MEL-3 and NRAS 1007). Nonetheless, we were not able to confirm this radiosensitization effect with clonogenic assays (Supplementary Figure S2). This could be explained by the selection of MEKi-resistant cells and/or the loss of apoptotic cells initiated after spheroid dissociation prior to cell seeding. However, it should be noted that NRAS 1007 spheroids demonstrated very high radiosensitivity to [^131^I]ICF01012, with the complete extinction of clonogenic survival for spheroids treated with TRT (compared to control: *p <* 0.0001, Supplementary Figure S2). The low apoptotic ratios with TRT are probably linked to the fact that TRT efficiency relies on mitotic catastrophe [18], characterized by a phase G2/M cell-cycle arrest. Cell-cycle arrest was indeed observed for SK-MEL-3 cells treated by TRT (Supplementary Figure S3), whereas a decrease in the S-phase was observed with MEKi treatment. The association of MEKi and TRT decreased the G2/M arrest due to fewer proliferating cells in S phase.

### 3.2. MEKi Inhibits [^131^I]ICF01012-Induced ERK1/2 Phosphorylation in BRAF- and NRAS-Mutant Melanoma Spheroid Models

**Figure 2:**
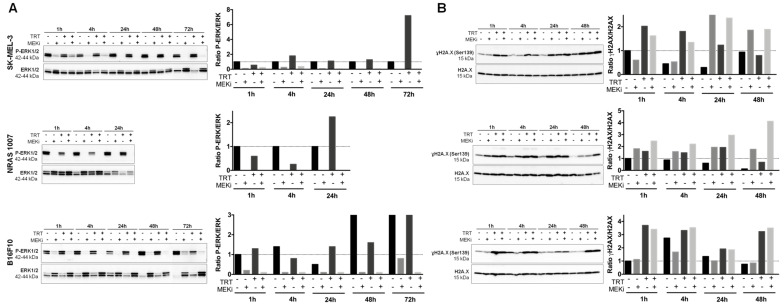
Quantification of p-ERK expression and analysis of DNA damage in melanoma spheroids following treatment with [^131^I]ICF01012-TRT, MEKi, or both. (**A**) Western blot analysis of P-ERK1/2 and ERK1/2 of total cellular protein extracted from SK-MEL-3, NRAS 1007, and B16F10 spheroids treated or not with TRT, MEKi, or both for the indicated times. The density of each P-ERK1/2 band was corrected using the density of the corresponding total ERK1/2 band. All values were normalized to the control (1 h). (**B**) The expression of phospho-H2A.X (γH2A.X) and total H2A.X were analyzed by Western blotting for SK-MEL-3, NRAS 1007, and B16F10 spheroids treated or not with TRT, MEKi, or both for the indicated times. The density of each γH2A.X band was corrected using the density of the corresponding total H2A.X band. For Western blot, spheroids from 3 different experiments were pooled. In graphs, y-scales are not identical between cell lines.

We assessed the levels of constitutive and [^131^I]ICF01012-induced phosphorylated ERK1/2 (p-ERK) by Western blot analysis. All spheroids displayed high basal levels of p-ERK. For SK-MEL-3 and 1007 spheroids, such high basal p-ERK levels are likely due to BRAF and NRAS mutations. B16F10 spheroids are wild-type for BRAF and NRAS. Thus, the high basal level of p-ERK is most likely due to the 3D spheroid cell-culture model. Indeed, it has been described that cell-cell contacts and interactions between cells are enhanced by the 3D spatial organization of the cells, which influences the signal transduction pathways and biological functions [43]. This hypothesis was confirmed by comparing the basal p-ERK expression in B16F10 spheroids to that of cells grown in 2D monolayer culture system (Supplementary Figure S4). In addition, it has been shown that increased cell-cell contact or hypoxia could activate the MAPK pathway [44]. TRT treatment of SK-MEL-3 spheroids slightly increased the level of p-ERK protein at 4 h and then at 72 h post-irradiation (Figure 2A). However, treatment with MEKi resulted in complete and maintained inhibition of constitutive and TRT-induced p-ERK. Treatment of NRAS 1007 spheroids with TRT increased p-ERK expression at 24 h post-irradiation, whereas MEKi alone or combined with [^131^I]ICF01012 completely inhibited constitutive and TRT-induced p-ERK expression (Figure 2A). This increase of p-ERK at 24 h post-irradiation could be explained by the high radiosensitivity of NRAS 1007 cells growing in spheroids, which could lead to cell death and selection of resistant cells. TRT did not increase p-ERK levels in B16F10 spheroids, except for a slight increase at 24 h post-irradiation (Figure 2A).

### 3.3. MEKi Enhances the Radiosensitivity of BRAF- and NRAS-Mutant Melanoma Spheroids, Leading to Increased DNA Double-Strand Breaks

We investigated whether the radio-sensitizing effect of MEKi was also due to DNA damage by performing Western blot analysis of the phosphorylated form of H2A histone family member X (γH2A.X) protein levels. The irradiation of SK-MEL-3 spheroids by [^131^I]ICF01012 induced an increase in the level of γH2A.X Serine 139 (Ser139) protein, indicating an increase in DNA double-strand breaks from 1 to 48 h post-irradiation (Figure 2B). Combining MEKi with TRT induced an increase in the level of γH2A.X(Ser139) after 24 (1.1-fold increase) and 48 h (1.1-fold increase) of irradiation relative to that with TRT alone. Treatment of SK-MEL-3 spheroids with MEKi alone also increased the level of γH2A.X at 24 and 48 h post-treatment. This can be explained by the induction of apoptosis in these spheroids and confirms the results obtained with the apoptosis assay and Western blot analysis of the cleaved PARP-1 protein (Figure 1A,B). Combining MEKi and TRT enhanced the level of γH2A.X(Ser139) in NRAS 1007 spheroids after 1 h of irradiation (0.8-fold increase) relative to TRT treatment alone and they remained higher up to 48 h post-irradiation (3.4-fold increase) (Figure 2C). In accordance with our previous data [24], [^131^I]ICF01012-TRT alone induced increased levels of γH2A.X in B16F10 spheroids. However, combining MEKi with TRT failed to radio-sensitize BRAF and NRAS wild-type B16F10 spheroids (Figure 2B).

### 3.4. [^131^I]ICF01012 Accumulates in NRAS 1007 Tumors, with a Favorable Dosimetry for TRT

**Figure 3:**
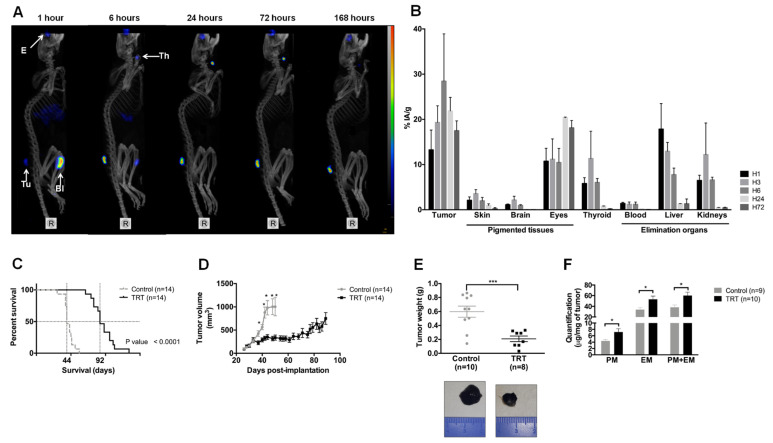
[^131^I]ICF01012 biodistribution and efficiency in an NRAS-mutant murine model. Results are presented as mean +/− SD (**D**,**F**) and as median, 1st quartile, and 3rd quartile (**E**). (**A**) Maximum intensity projection from SPECT-CT imaging of the same mouse from 1 h post-injection to 168 h shows tumor (Tu), eyes (E), thyroid (Th), and bladder (Bl) fixation of [^131^I]ICF01012. (**B**) Biodistribution of [^131^I]ICF01012, expressed as the percentage of injected activity/gram, was determined for each timepoint and each non-target organ by activity count (n = 3 mice per timepoint). (**C**,**D**) Survival curve (**C**) and tumor growth (**D**) were determined using 14 animals for each group. (**E**) Tumor weight for 10 control mice and eight TRT-treated mice, sacrificed 10 days after treatment, illustrated by 2 tumors representative of observed differences between control and TRT tumors. (**F**) Pheomelanin (PM) and eumelanin (EM) were quantified for nine control tumors and 10 TRT tumors. (* *p* < 0.05, *** *p* < 0.001).

SPECT-CT imaging (Figure 3A) showed rapid and persistent accumulation of [^131^I]ICF01012 in tumors, which was maximal at 6 h post-injection. As expected, non-saturated thyroid showed intense fixation from 6 to 72 h post-injection. Specific [^131^I]ICF01012 fixation was observed in the eyes, which contain both types of melanin. One hour after injection, [^131^I]ICF01012 accumulated in the bladder and was completely eliminated after 24 h. The biodistribution in the tumor showed rapid and persistent accumulation of [^131^I]ICF01012 over 72 h (16.1% IA/g) (Figure 3B), which was maximal at 6 h post-injection (28.5% IA/g).

We determined the absorbed dose for NRAS 1007 tumors and non-targeted organs from biodistribution studies (Figure 3B and Supplementary Table S3) after i.v. injection of 0.37 MBq [^131^I]ICF01012 and calculated the dosimetry for certain organs using *S*-values (Supplementary Table S4). The dose used for the therapeutic injection (18.5 Gy) showed high delivery of [^131^I]ICF01012 to the tumor (93.2 Gy) relative to the other organs. A low absorbed dose was deposited in excretion organs, such as the kidneys and liver (1.8 Gy and 4.3 Gy, respectively). The absorbed dose for the thyroid was very high (228.1 Gy), as potassium iodide was not used to block iodine absorption by the thyroid. As we used C57BL/6 mice, the dose delivered to the pigmented eyes was also very high (137.5 Gy), as expected.

An injected activity of 3.7 GBq is classically considered as a standard therapeutic dose in human iodine-TRT protocols. Based on this activity, [^131^I]ICF01012 dosimetry for humans extrapolated using Olinda *S*-values (Table 1) did not show toxic levels for the studied organs. Total body irradiation was evaluated to be 0.021 Gy.

### 3.5. [^131^I]ICF01012 Induces Significant Tumor Regression and Extended Survival for Mice Bearing NRAS 1007 Tumors

[^131^I]ICF01012 significantly prolonged the survival of mice bearing NRAS mutant tumors relative to the control group (Figure 3C, median survival: 92 vs. 44 days, *p* < 10^−4^). This was associated with slowed tumor growth from day 5 post-[^131^I]ICF01012 injection (Figure 3D). The doubling time of treated tumors was five-times longer than that of the control group (DT = 26.23 vs. 5.177 days^−1^, *p* < 10^−4^). Ten days post-[^131^I]ICF01012 treatment, the tumor weight was significantly lower in the treated group (Figure 3E, 0.208 vs. 0.598 g, *p* = 7 × 10^−4^). Consistent with our previous finding using SK-MEL-3 tumors [24], [^131^I]ICF01012-TRT induced a significant 1.5-fold increase of both pheomelanin (control: 4.39 vs. TRT: 7.19 µg/mg) and eumelanin (33.97 vs. 53.05 µg/mg) levels (Figure 3F). However, the proportion of pheomelanin (11.71% vs. 12.52%) and eumelanin (88.29% vs. 87.48%) positive tumors was similar for control and TRT-treated animals, respectively. Interestingly, we did not evidence significant loss of weight, alterations of general condition, vitiligo, or changes in behavior that could suggest any ocular toxicity.

### 3.6. [^131^I]ICF01012 Reduces Lymph-Node Metastases in the NRAS 1007 Model

**Figure 4:**
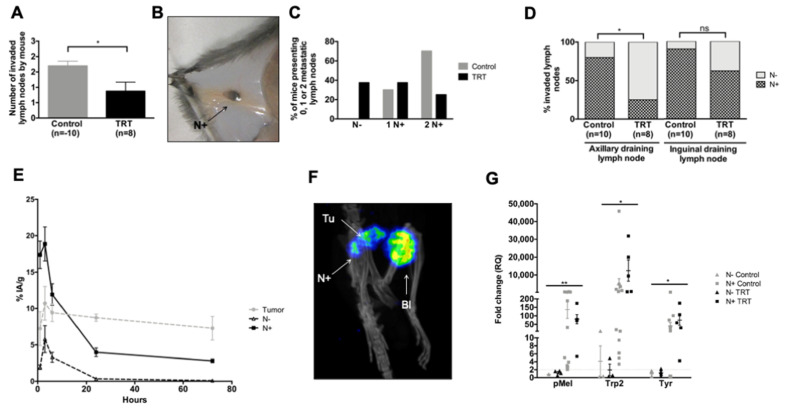
[^131^I]ICF01012 decreases lymph-node dissemination. Results are presented as mean +/− SD (**A**,**E**) and as median, 1st quartile, and 3rd quartile (**G**). (**A–E**) The numbering and topographic location of metastatic LNs (N+) were determined for each mouse 10 days after treatment (10 control, 8 TRT): number of N+ per mouse (**A**), typical N+ (**B**), proportion of mice presenting 0 (N−), 1, or 2 N+ (**C**)**,** repartition of invaded LNs (**D**). The radioactivity present in (T) tumors and (N+) (**E**) was determined during biodistribution studies on three mice per timepoint (1, 3, 6, 24, and 72 hours after injection): 24 LNs were macroscopically metastatic (N+) and 32 were non-metastatic (N−). (**F**) Visualization of an N+ by SPECT-CT imaging (T: tumor, N: lymph node, Bl: bladder). (**G**). Trp2, pMel, and Tyr gene expression in axillary and inguinal draining LNs was measured by RT-qPCR in 18 N+ (control group: n = 13 and TRT group: n = 5) and 7 N− (control group: n = 3 and TRT group: n = 4). LNs were considered to be metastatic when they showed a ≥ 2-fold change for at least two genes. (* *p* < 0.05, ** *p* < 0.01).

We observed macroscopic melanin-pigmented LN metastases. [^131^I]ICF01012 treatment decreased the number of macroscopically invaded LNs in mice (Figure 4A,B: 0.875 vs. 1.70, *p* = 0.0310). Indeed, all mice presented at least one metastatic LN in the control group, whereas 37.5% of the mice in the TRT-treated group did not (Figure 4C). The proportion of mice with two invaded LNs was also higher in the control group (Figure 4C, 70% vs. 25%). Concerning the topography, 62% of inguinal and 25% of axillary right LNs were metastatic in the treated mice versus 90% and 80% in the control group, respectively (Figure 4D, *p* = 0.0306). Assessment of the biodistribution and SPECT-CT imaging (Figure 4E,F) confirmed that [^131^I]ICF01012 was present in the LNs. [^131^I]ICF01012 uptake was significantly higher in metastatic LNs, which was maximal at 6 h post-[^131^I]ICF01012 injection, corresponding to the highest uptake of the tumor and confirming the presence of melanin in the metastatic LNs. We assessed specific molecular melanoma markers by RT-qPCR analysis to assess the presence of melanoma cells in the LNs [45]. The expression of melanin biosynthesis-related genes, such as premalenosome gene (*pMel*), dopachrome tautomerase (*Trp2 (Dct)*), and *tyrosinase (Tyr)* was significantly higher in metastatic LNs (Figure 4G) (*p* = 0.0062, *p* = 0.0428, *p* = 0.0115, respectively). The levels of mRNA corresponding to these genes in the metastatic LNs were similar between the TRT-treated and control groups. These results provide evidence for [^131^I]ICF01012 uptake by metastatic LNs and its efficiency to reduce the spread of melanoma throughout the lymphatic system.

### 3.7. [^131^I]ICF01012 Modifies Oxidative Stress, Inflammatory, and P53 Signatures

**Figure 5:**
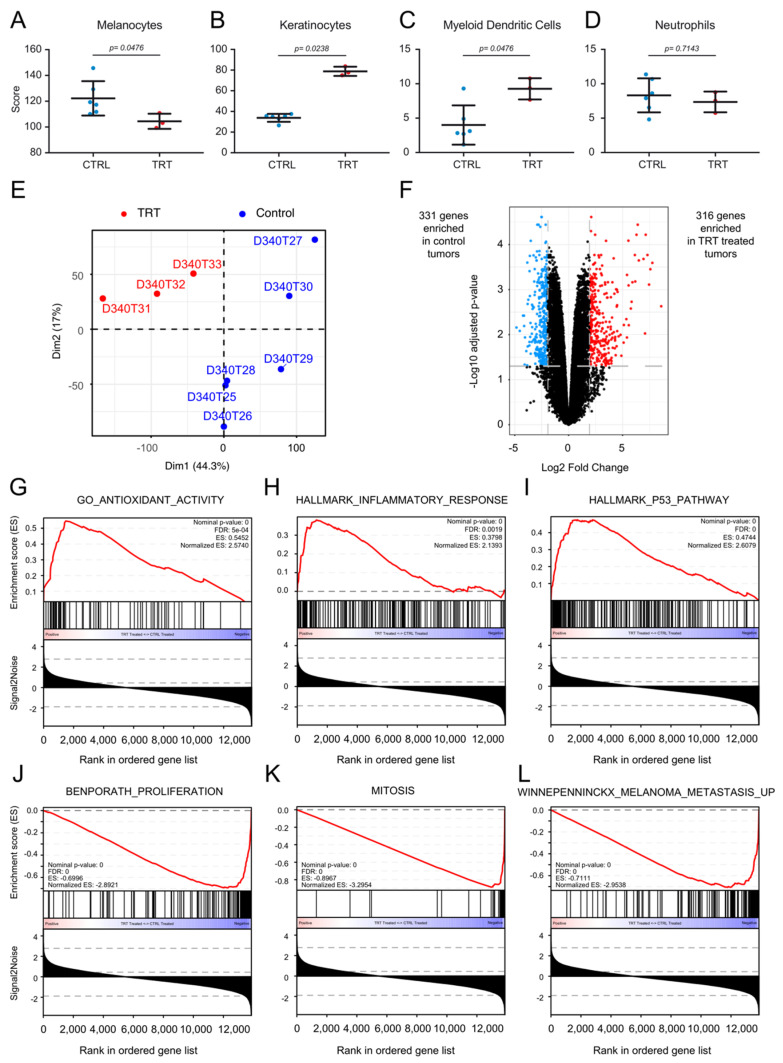
Bioinformatics analysis of treated and non-treated tumors. (**A**–**D**) Deconvolution results comparing the enrichment of various cell populations between six control-treated tumors and three TRT-treated tumors. Results for the three significantly enriched cell populations: melanocytes (**A**), keratinocytes (**B**), and myeloid dendritic cells (**C**) and one of the non-modified populations, (neutrophils) (**D**)**,** are depicted. (**E**) Principal component analysis (PCA) showing a clear demarcation between TRT-treated tumors and mock-treated tumors. (**F**) Volcano-plot depicting the differential analysis results between TRT-treated tumors and control tumors. Differentially expressed genes were defined as genes with an adjusted *p*-value < 0.05 and a fold-change > 2 SD of the fold change (https://www.ncbi.nlm.nih.gov/geo/query/acc.cgi?acc=GSE162536) (accessed on June 2020). In total, 316 genes were enriched in TRT-treated tumors vs. 331 in mock-treated tumors. An interactive volcano plot is presented in Supplementary Figure S6. (**G**–**L**) Gene-set enrichment results from gene-set enrichment analysis (GSEA). The gene sets enriched in TRT-treated tumors were related to antioxidant activity (**G**), an inflammatory response (**H**)**,** and activation of the P53 pathway (**I**). The gene sets enriched in control tumors were related to proliferation (**J**), mitosis (**K**)**,** and melanoma metastasis (**L**).

We next performed transcriptomic analysis of TRT-treated and control tumors. First, deconvolution analyses showed the TRT-treated tumors to contain fewer melanocytes (Figure 5A) and more keratinocytes (Figure 5B) than control tumors, demonstrating the antitumoral activity of [^131^I]ICF01012. Intriguingly, we observed a significant enrichment of dendritic cell-related genes in TRT-treated tumors relative to the control group (Figure 5C). These modifications were not present for other cell types, such as, for example, neutrophils, (Figure 5D and Supplementary Figure S5). Principal component analysis (PCA) showed that TRT-treated tumors form a significant cluster (*p* = 0.014) separate from the controls (Figure 5E) as PCA plots (Supplementary Video S1). We performed differential analysis to compare TRT-treated and control tumors. In total, 316 genes were upregulated and 331 downregulated in TRT-treated vs. control tumors with a fold-change > 2SD and an adjusted *p*-value < 0.05 (Figure 5F).

An interactive volcano plot is presented in Supplementary Figure S6. The differentially expressed genes are also listed in Supplementary Table S5. Pathway and ontology enrichment analysis by over-representation were conducted on the BioPlanet (BioPlanet 2019) and Gene Ontology (GO-Biological Pathways 2018) databases, respectively, using the 316 and 331 differentially expressed genes. The 331 genes enriched in the control tumors mapped to pathways and ontologies involved in the cell cycle, cell proliferation, and cell replication (Supplementary Figure S7A,B). Interestingly, the 316 genes significantly overexpressed in TRT-treated tumors mapped to pathways related to lipid and folate metabolism (Supplementary Figure S7C). The ontologies enriched in TRT-treated tumors were related to lipid metabolism and the presence of more keratinocytes in the samples (Supplementary Figure S7D). Gene-set enrichment analysis (GSEA) was conducted to document the mechanism of action of [^131^I]ICF01012. The results suggest the involvement of oxidative stress pathways (Figure 5G) and inflammation (Figure 5H) and activation of the p53 signaling pathway (Figure 5I). The gene sets enriched in the control tumors reflected the effect observed during the follow-up of tumor growth in mice, with an enrichment of proliferation (Figure 5J) and mitosis (Figure 5K) signatures. A signature of metastatic melanoma was also significantly enriched in control tumors relative to TRT tumors (Figure 5L). These bioinformatics data indeed correlate perfectly with our in vivo observations.

## 4. Discussion

MAPK pathway activation has been shown to play a role in external beam radiation therapy (EBRT) radioresistance [46] but is still poorly documented in TRT. We have previously shown that [^131^I]ICF01012 induces an initial decrease in p-ERK levels and then a non-significant increase in a B16BL6 model, suggesting potential radioresistance mechanisms [18]. Here, we evaluated the potential of combining TRT with an MEK inhibitor to overcome MAPK-mediated radioresistance in a panel of melanoma 3D spheroid models that mimic tumor architecture. We used the SK-MEL-3 (BRAF^V600E^) and NRAS 1007 (NRAS^Q61K^) cell lines, which exhibit a constitutively activated MAPK pathway, and the B16F10 cell line (wild-type BRAF/NRAS).

Here, we show that combining TRT with MEKi can overcome such activation by reducing ERK phosphorylation in BRAF- and NRAS-mutant melanoma. In addition, the inhibition of ERK phosphorylation significantly enhanced apoptosis in BRAF- and NRAS-mutant melanoma spheroids, but not the wild-type BRAF/NRAS spheroid model. However, combining MEKi with TRT did not modify the clonogenic capacity and subsequently the survival fraction of BRAF mutant spheroids. Such a residual survival fraction may indicate that the process selected a number of MEKi- and/or TRT-resistant cells. However, colony forming assay showed that [^131^I]ICF01012-TRT alone was able to induce complete inhibition of the clonogenic survival capacity of NRAS 1007 cells, highlighting their high radiosensitivity. Furthermore, aside from the total melanin content being similar (Supplementary Figure S8B), [^131^I]ICF01012 uptake by NRAS 1007 spheroids was significantly lower than that of B16F10 spheroids (Supplementary Figure S8A), reinforcing the hypothesis of the intrinsic radiosensitivity of these cells. Complementary studies on other NRAS mutant melanoma cell lines are required to show that such high TRT radiosensitivity is related to NRAS mutation, although previous EBRT studies have reported increased radiosensitivity in vitro [47] and in vivo [48] when NRAS is mutated. As expected, combining MEKi with TRT had no effect on the clonogenic capacity or apoptosis of BRAF and NRAS wild-type B16F10 spheroids.

It has been shown that MEKi (trametinib)-induced EBRT radiosensitization is related to the acquisition of senescence and perturbation of the cell cycle, with a decrease in the proportion of cells in S phase and a blockade in the G1 phase [25]. However, no effect on apoptosis, delayed DNA damage repair, or mitotic catastrophe has been described. Our results demonstrate that combining MEKi with TRT induces a similar decrease in the proportion of cells in S phase in BRAF spheroids and an increase in DNA double-strand breaks in NRAS spheroids. However, we showed that combining MEKi with TRT induces a marked increase in apoptosis of BRAF- and NRAS-mutant melanoma cells, which could be specific to TRT irradiation or related to the spheroid 3D architecture. These results suggest that the addition of MEKi to TRT radio-sensitizes melanoma cells in our spheroid model by increasing DNA damage and apoptosis in BRAF- and NRAS-mutant cells. Although the spheroids mimic 3D tumor architecture, this model does not fully reflect the complexity of the tumor with neoangiogenesis and the active microenvironment (including cancer-associated fibroblast and the immune infiltrate), components that may impact the MAPK signaling pathway. The observed radio-sensitization in BRAF- and NRAS-mutant melanoma cells lines treated with TRT and MEKi combination will need, in the future, to be assessed by in vivo studies using human BRAF- and NRAS-mutant melanoma xenografts. The use of MAPK inhibitors to radio-sensitize tumors to TRT appears to be a promising strategy in BRAF- and NRAS-mutant melanoma, although the lack of an in vivo study to confirm the results is the main limitation of this work.

We used the syngeneic NRAS^Q61K^ 1007 model [28], which mimics the human NRAS-mutant model [49], to address the efficiency of [^131^I]ICF01012-TRT in this pigmented melanoma. NRAS^Q61K^ 1007 tumors clearly accumulate a sufficient amount of [^131^I]ICF01012 to improve the efficiency of TRT, with an absorbed dose in the supra-therapeutic range (94 Gy), as the curative dose for EBRT is 50 to 60 Gy. The extrapolated dosimetry to humans does not suggest major toxicity, as the absorbed doses of non-target organs remained within the recommended limits, as previously reported for extrapolated doses from a rabbit bio-distribution study [50]. The main concern with TRT using [^131^I]ICF01012 is potential ocular toxicity due to a high absorbed dose related to [^131^I]ICF01012 fixation on the melanin present in the choroid and retinal pigment epithelium. However, [^131^I]ICF01012 rabbit dosimetry extrapolated to humans has shown that the absorbed dose in the eyes remains acceptable [50]. Furthermore, the first clinical trial using a similar radiolabeled melanin-binding molecule showed clear clinical improvement for 2 of 5 patients with metastatic melanoma and did not report any ocular toxicity [51]. In the present NRAS-mutant melanoma model, [^131^I]ICF01012-TRT dramatically increased mouse survival and reduced tumor growth. These observations clearly have to be confirmed using other NRAS-mutant tumors. There are several possible explanations for such high radiosensitivity compared to our previous results on B16 tumors [18,20,21]. One is that the proportion of pheomelanin in NRAS mutant tumors (11.67% in the control tumors) is clearly higher than that in the B16BL6 tumor (1.61%) [33], which could considerably increase TRT-induced oxidative stress [52]. Previously, TLDA-analysis in B16BL6 tumors showed a significant increase in antioxidant enzyme expression (GPx1, heme oxygenase, SOD3) in treated tumors three days post-TRT [18]. This is fully supported by the present transcriptomic analysis, which emphasizes the increase in antioxidant gene expression in treated vs. control tumors. It could also be related to the enhancement of immunogenicity by radiation, as we already demonstrated for the B16F10 melanoma model [53], also consistent with the increased expression of inflammatory genes shown by transcriptomics. Additional studies need to be conducted to confirm that these pathways are involved in the NRAS^Q61K^ 1007 response to [^131^I]ICF01012-TRT, as modified lipid metabolism has already been reported to alter the efficiency of radioimmunotherapy [54]. In previous studies, we showed that TRT reduces hematogenous dissemination, especially lung invasion [21], by modifying pseudo-epithelial-mesenchymal transition pEMT-mechanisms [24]. Here, we demonstrate, for the first time, that [^131^I]ICF01012 penetrates the lymphatic system and limits LN metastases. Transcriptomic analyses also support the reduction of the metastatic capacity of melanoma by [^131^I]ICF01012-TRT.

## 5. Conclusions

In conclusion, this study reinforces the potential of [^131^I]ICF01012-TRT in the management of pigmented metastatic melanoma, especially its capacity to reduce metastatic dissemination. Future experiments that aim to clarify the mechanisms underpinning such high radiosensitivity to [^131^I]ICF01012-TRT will likely lead to a specific clinical trial including patients harboring NRAS metastatic melanoma, following the ongoing dose-escalation phase 1 study MELRIV1 (NCT03784625).

## Figures and Tables

**Table 1:** Extrapolated [^131^I]ICF01012 dosimetry for humans: absorbed dose (Gy/MBq) and dose (Gy) for 3.7 GBq extrapolated using Olinda *S*-values.

Organ/Tissue	Absorbed Dose (Gy·GBq^−1^)	Dose for 3.7 GBq (Gy)
Adrenals	0.006	0.023
Brain	0.007	0.025
Gall bladder	0.010	0.037
Lower large intestine	0.021	0.079
Small Intestine	0.048	0.176
Stomach	0.043	0.159
Upper large intestine	0.021	0.078
Heart	0.012	0.044
Kidneys	0.055	0.203
Liver	0.064	0.238
Lungs	0.028	0.105
Muscle	0.003	0.009
Ovaries	0.007	0.026
Pancreas	0.008	0.028
Red Marrow	0.003	0.010
Skin	0.001	0.004
Spleen	0.062	0.229
Testes	0.012	0.044
Thymus	0.002	0.007
Thyroid	0.044	0.163
Urinary Bladder	0.184	0.682
**Total body**	**0.006**	**0.021**

## Data Availability

The data generated by the transcriptomic study (Table S3) have been deposited in the Gene Expression Omnibus of the NCBI and are accessible through GEO Series accession number GSE GSE162536 (https://www.ncbi.nlm.nih.gov/geo/query/acc.cgi?acc=GSE162536). This series contains the raw reads (fastq) and aligned-reads (BAM) of nine tumors (3 treated and 6 non-treated), as well as the gene expression read counts and TPM values. The other data presented in this study are available on request from the corresponding author.

## Supplementary Materials

The following are available online at https://www.mdpi.com/2072-6694/13/6/1421/s1, Figure S1: Determination of the optimal dose of MEKi (Cobimetinib and GDC-0623) for the treatment of ^V600E^BRAF SK-MEL-3, ^Q61K^NRAS 1007, and ^WT^BRAF/NRAS B16F10 melanoma spheroids. Figure S2: Colony formation (n = 3) on melanoma spheroids after treatment with [^131^I]ICF01012-TRT, MEKi, or both. Figure S3: Cell-cycle analysis of SK-MEL-3 spheroids treated with TRT alone or in combination with MEKi. Figure S4: Western blot analysis of P-ERK1/2 and ERK1/2 of total cellular protein extracted from B16F10 cells that have been cultured in a 2D monolayer culture system. Figure S5: Deconvolution analyses of major immune cells subset. Figure S6: Interactive volcano plot depicting the differential analysis results between TRT treated tumors and Control tumors (see HTML file). Figure S7: Pathways and gene ontology analyses. Figure S8: [^131^I]ICF01012 uptake by spheroids and melanin content in spheroids. Table S1: Mice weight and tumor volume at start of experiment and endpoint for survival and mechanistical studies. Table S2: Primer sequences. Table S3: [^131^I]ICF01012 biodistribution in non-target organs in mice. Table S4: [^131^I]ICF01012 dosimetry in mice. Table S5: Differential mRNA expression analysis of treated vs. non-treated tumors. Video S1: Principal component analysis (PCA) of the three first dimensions of treated and non-treated tumors (see mp4 file).
