# Supplementary material for: Efficacy of Targeted Radionuclide Therapy Using [131I]ICF01012 in 3D Pigmented BRAF- and NRAS-Mutant Melanoma Models and In Vivo NRAS-Mutant Melanoma
Source: Cancers (Basel). 2021 Mar 20;13(6):1421. doi: 10.3390/cancers13061421 (PMC8003594; doi:10.3390/cancers13061421)
Supplement: Supplementary file 1 [file cancers-13-01421-s001.zip › Supplementary Material/Akil et al Cancers Supplementary Figures and Tables v3.pdf]

SUPPLEMENTARY DATA

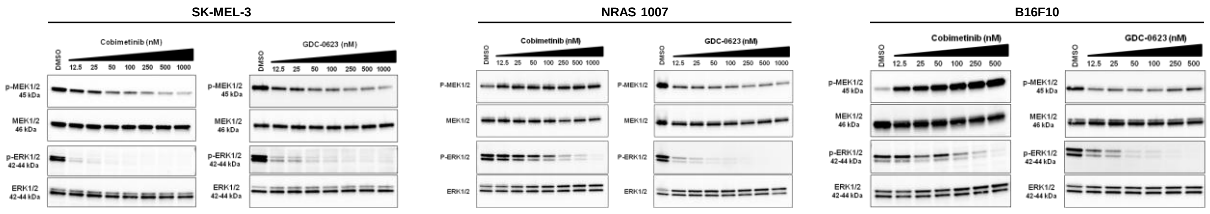

Figure S1. Determination of the optimal dose of MEKi (Cobimetinib and GDC-0623) for the treatment of ^{V600E}BRAF SK-MEL-3, ^{Q61K}NRAS 1007, and ^{WT}BRAF/NRAS B16F10 melanoma spheroids. The expression of phospho-MEK1/2 (P-MEK1/2), total MEK (MEK1/2), phospho-ERK1/2 (P-ERK1/2), and total ERK (ERK1/2) were analyzed by western blotting of total cellular protein extracted from spheroids treated for 24 h with the indicated concentrations of MEKi. The dose-response to two allosteric MEK inhibitors, cobimetinib and GDC-0623, was analyzed on a panel of melanoma 3D spheroid culture models bearing different genetic mutations: SK-MEL-3 (^{V600E}BRAF), NRAS 1007 (^{Q61K}NRAS), and B16F10 (BRAF/NRAS wild-type). MEKi treatment of NRAS 1007 spheroids, as well as the BRAF/NRAS wild-type spheroids, was expected to increase the phosphorylation level of MEK (pMEK) through feedback-mediated RAF kinase activity (44). Indeed, cobimetinib treatment of NRAS 1007 and B16F10 spheroids induced an increase in pMEK levels, but not GDC-0623. Both MEKi reduced the level of pMEK in SK-MEL-3 BRAF-mutant spheroids, with a slightly higher efficiency for cobimetinib.

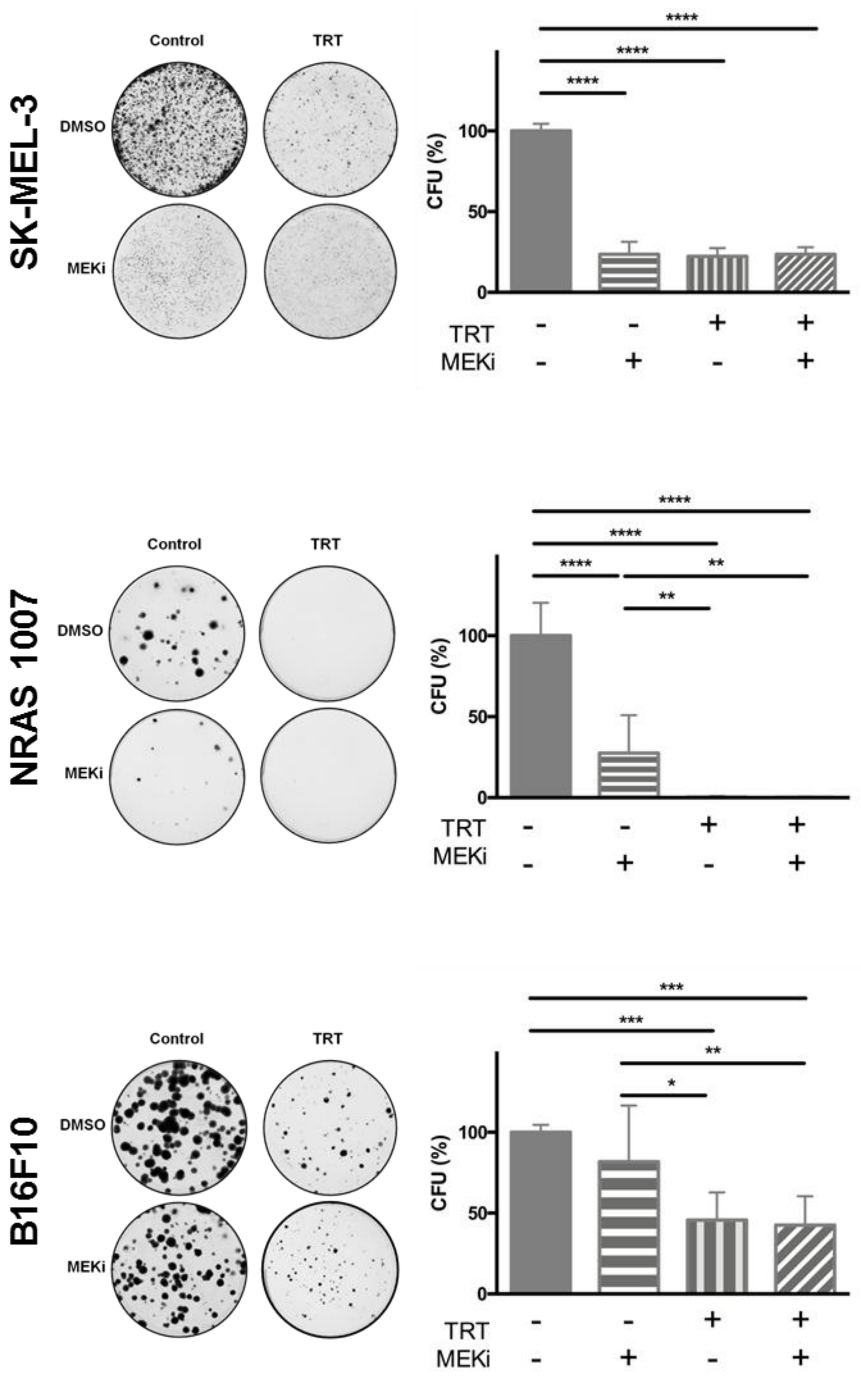

Figure S2. Colony formation (n=3) on melanoma spheroids after treatment with $[^{131}I]$ICF01012-TRT, MEKi, or both. Results are presented as Mean +/- SD. Dissociated cells from SK-MEL-3, NRAS 1007, and B16F10 spheroids were seeded for colony formation assays 24 h after treatment and stained with crystal violet on days 18, 15, and 8, respectively. The SK-MEL-3 colony formation assay (Figure 1A) showed that MEKi (20.22%, $p<0.0001$) and TRT (18.12%, $p<0.0001$) significantly decreased the clonogenic surviving fraction relative to control spheroids. However, compared to TRT- and MEKi-monotherapy groups, we did not show a greater decrease when MEKi and TRT were combined (22.08%, compared to control spheroids: p<0.0001, compared to MEKi: p>0.9999 and to TRT: p=0.9660). NRAS 1007 spheroids demonstrated very high radiosensitivity to $[^{131}I]$ICF01012, with the complete extinction of clonogenic survival for spheroids treated either with TRT alone or TRT + MEKi (for both conditions, compared to control: $p<0.0001$, Figure 1A). Treatment of NRAS 1007 spheroids

with MEKi alone induced a significant decrease in the surviving fraction compared to the untreated control group (27.61%, $p<10^{-4}$), whereas the same treatment of B16F10 spheroids (Figure 1A) did not significantly decrease the clonogenic surviving fraction relative to that of control spheroids (81.82%, p=0.3277). Treatment of B16F10 spheroids with TRT or TRT + MEKi led to a similar decrease in the surviving fraction relative to that of controls (45.71% ($p<10^{-3}$) and 42.54% ($p<10^{-4}$), respectively).

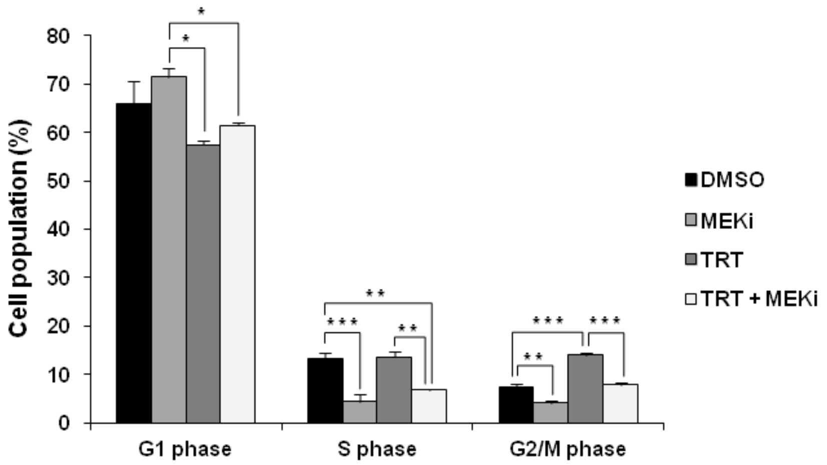

Figure S3. Cell-cycle analysis of SK-MEL-3 spheroids treated with TRT alone or in combination with MEKi. This analyse was performed on 3 different 3D culture according to protocol indicated in Material and Methods section. (*p<0.05, **p<0.01, ***p<0.001, ****p<0.0001).

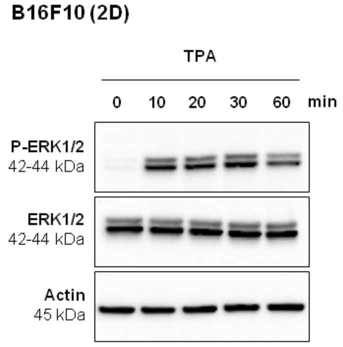

Figure S4. Western Blot analysis of P-ERK1/2 and ERK1/2 of total cellular protein extracted from B16F10 cells that have been cultured in a 2D monolayer culture system. The cells were treated or not with TPA (12-O-Tetradecanoylphorbol-13-Acetate) for 10 to 60 min.

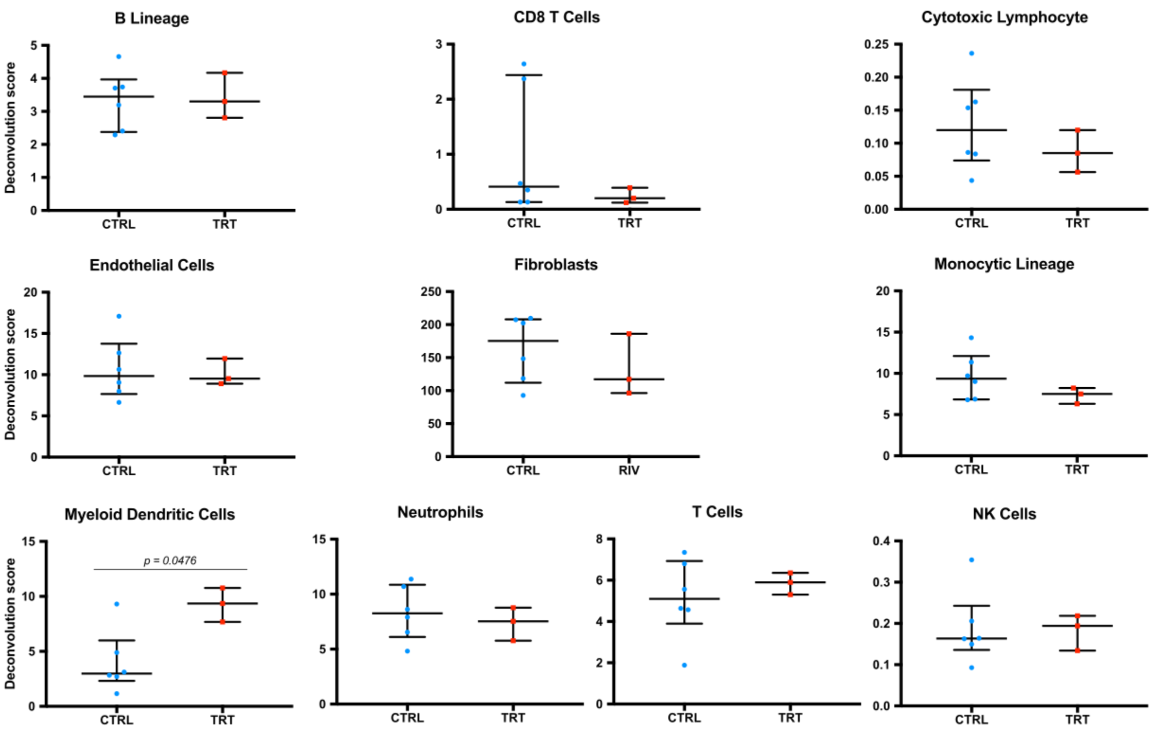

Figure S5. Deconvolution analyses of major immune cells subset.

Figure S6. Interactive volcano plot depicting the differential analysis results between TRT treated tumors and Mock-Treated tumors (see HTML file).

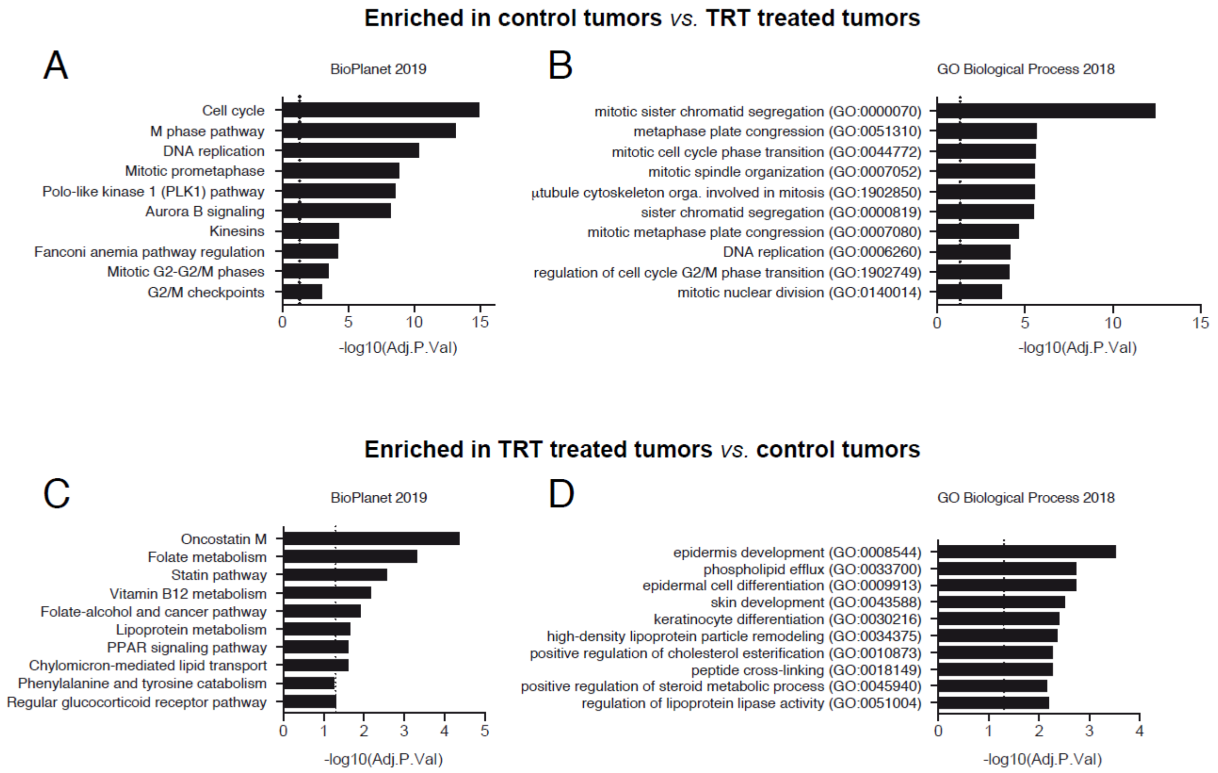

Figure S7. Pathways and gene ontology analyses

Pathways and ontologies found to be enriched by over-representation analysis of genes enriched in TRT-Treated and mock-Treated melanoma, respectively. Top 10 over-represented pathways from BioPlanet (2019) **(A)**, and top 10 biological process ontologies from Gene Ontology (GO – BP, 2018) **(B)** for mock-treated melanoma. Top 10 over-represented pathways from BioPlanet (2019) **(C)** and top 10 biological process ontologies from Gene Ontology (GO – BP, 2018) **(D)** for TRT-treated melanoma.

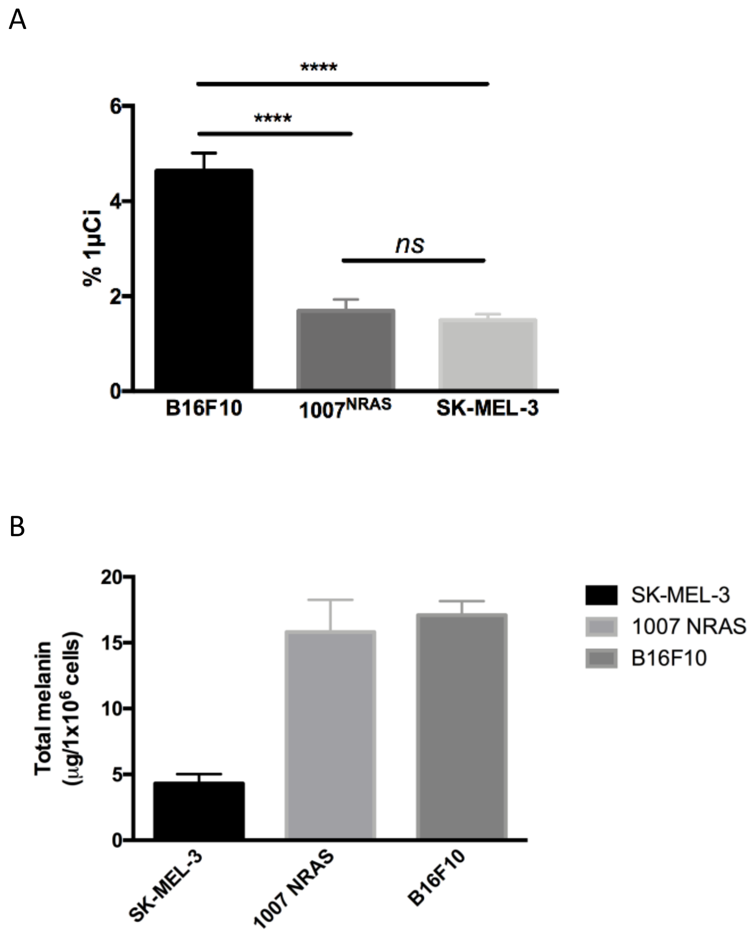

Figure S8. [131I]ICF01012 uptake by spheroids (A) and **melanin content** in spheroids (**B).**

Table S1 – Mice weight and tumor volume at start of experiment and endpoint for survival and mechanistical studies.

			**Day of injection**	**Endpoint** *(for survival study: tumor volume approximatively 1500mm^3)* *(for mechanistical study: 10 days after injection)*
**Survival Study**	**Control**	**Mean tumor volume**	260.36mm3	1566.74mm3
		**Median tumor volume**	243.91mm3	1547.80mm3
		**Mean mice weight**	26.8g	27.9g
		**Median mice weight**	26.6g	27.8g
	**TRT**	**Mean tumor volume**	283.91mm3	1434.55mm3
		**Median tumor volume**	251.68mm3	1483.05mm3
		**Mean mice weight**	27.2g	26.5g
		**Median mice weight**	27.6g	26.4g
**Mechanistical Study**	**Control**	**Mean tumor volume**	237.84mm3	698.90mm3
		**Median tumor volume**	230.34mm3	725.49mm3
		**Mean mice weight**	28.9g	28.9g
		**Median mice weight**	29.3	28.5g
	**TRT**	**Mean tumor volume**	255.39mm3	296.20mm3
		**Median tumor volume**	206.01mm3	269.68mm3
		**Mean mice weight**	29.4g	29.1g
		**Median mice weight**	29.7g	28.9g

Table S2. Primer sequences.

**Gene**	**NCBI Reference Sequence**	**Sequence of primers 5' to 3'**	**Amplicon size (bp)**	**Annealing temperature (°C)**
pmel	NM_021882.4	F: AAATGCCAACCACAGAGGTC<br>R: CAAGCATTATGGTGTCGGTG	246	60
Trp2	NM_010024.3	F: AGACTACGTGATCACCACGC<br>R: CAACAGATGGTACCTGTGCC	214	60
Tyr	NM_011661.5	F: CCATGCTTTTGTGGACAGTATTTT<br>R: CGATAGGTGCATTGGCTTCTG	89	60
Gapdh	NM_001289726.1	F: TGCGACTTCAACAGCAACTC<br>R: ATGTAGGCCATGAGGTCCAC	143	60

(F, forward primer; R, reverse primer)

Table S3 – [^{131}I]ICF01012 biodistribution in non-target organs in an NRAS-mutant murine model (n=3 mice per timepoint).

	Percentage of injected activity/gram (expressed as mean+/-SD)														
**Organs**	**H1**			**H3**			**H6**			**H24**			**H72**		
Small intestine	5,28	±	0,81	10,95	±	6,52	5,18	±	0,93	0,35	±	0,01	0,08	±	0,01
Cæcum	2,20	±	0,56	5,07	±	3,26	2,67	±	0,78	0,64	±	0,06	0,12	±	0,02
Colon	4,00	±	0,74	6,94	±	1,89	4,56	±	1,47	0,85	±	0,06	0,14	±	0,02
Heart	2,21	±	0,59	3,82	±	2,20	1,93	±	0,39	0,23	±	0,06	0,08	±	0,01
Lungs	5,75	±	2,34	9,89	±	4,91	5,22	±	1,60	0,31	±	0,01	0,11	±	0,02
Stomach	7,68	±	1,59	15,59	±	6,89	11,03	±	2,29	1,62	±	0,11	0,30	±	0,04
Spleen	10,87	±	6,01	14,84	±	4,96	11,96	±	6,97	0,19	±	0,01	0,06	±	0,00
Prostate	2,53	±	0,66	4,02	±	2,64	1,76	±	0,11	0,63	±	0,33	0,08	±	0,00
Testes	1,63	±	0,40	3,37	±	1,93	1,36	±	0,21	0,13	±	0,01	0,03	±	0,00
Bone	1,65	±	0,09	2,78	±	1,55	1,72	±	0,73	0,10	±	0,01	0,02	±	0,00
Muscle	0,89	±	0,23	1,44	±	0,47	0,64	±	0,24	0,05	±	0,01	0,02	±	0,00
Brown adipose tissue	2,23	±	0,55	3,59	±	2,24	1,50	±	0,15	0,11	±	0,01	0,05	±	0,01

Table S4- [^{131}I]ICF01012 dosimetry in mice: time-integrated activity (Bq.s/MBq) and absorbed dose (Gy/MBq) determined by biodistribution studies and the extrapolated doses (Gy) for 18.5MBq.

Organ/Tissue	Time-integrated activity (Bq.s/MBq)	Absorbed dose (Gy.MBq-1)	Dose for 18.5MBq (Gy)
**Tumor**	8.44×10^{10}	5.04	93.18
**Liver**	4.16×10^{9}	0.23	4.29
**Kidneys**	1.11×10^{9}	0.09	1.74
**Lungs**	3.49×10^{8}	0.05	0.85
**Thyroïd**	4.85×10^{8}	12.33	228.06
**Eyes**	1.05×10^{10}	7.43	137.46

Table S5. Differential mRNA expression analysis of treated *vs*. non-treated tumors.

The differential gene expression list for treated *vs.* non-treated tumors is given in the first sheet. The genes over-expressed for non-treated *vs.* treated tumors or over-expressed for treated *vs.* non-treated tumors are listed in the second and third sheets, respectively. (See Excel file)

Video S1. Principal component analysis (PCA) of the three first dimensions of treated and non-treated tumors (see mp4 file)
