# Supplementary figures and images for: Efficacy of Targeted Radionuclide Therapy Using [131I]ICF01012 in 3D Pigmented BRAF- and NRAS-Mutant Melanoma Models and In Vivo NRAS-Mutant Melanoma
Source: Cancers (Basel). 2021 Mar 20;13(6):1421. doi: 10.3390/cancers13061421 (PMC8003594; doi:10.3390/cancers13061421)

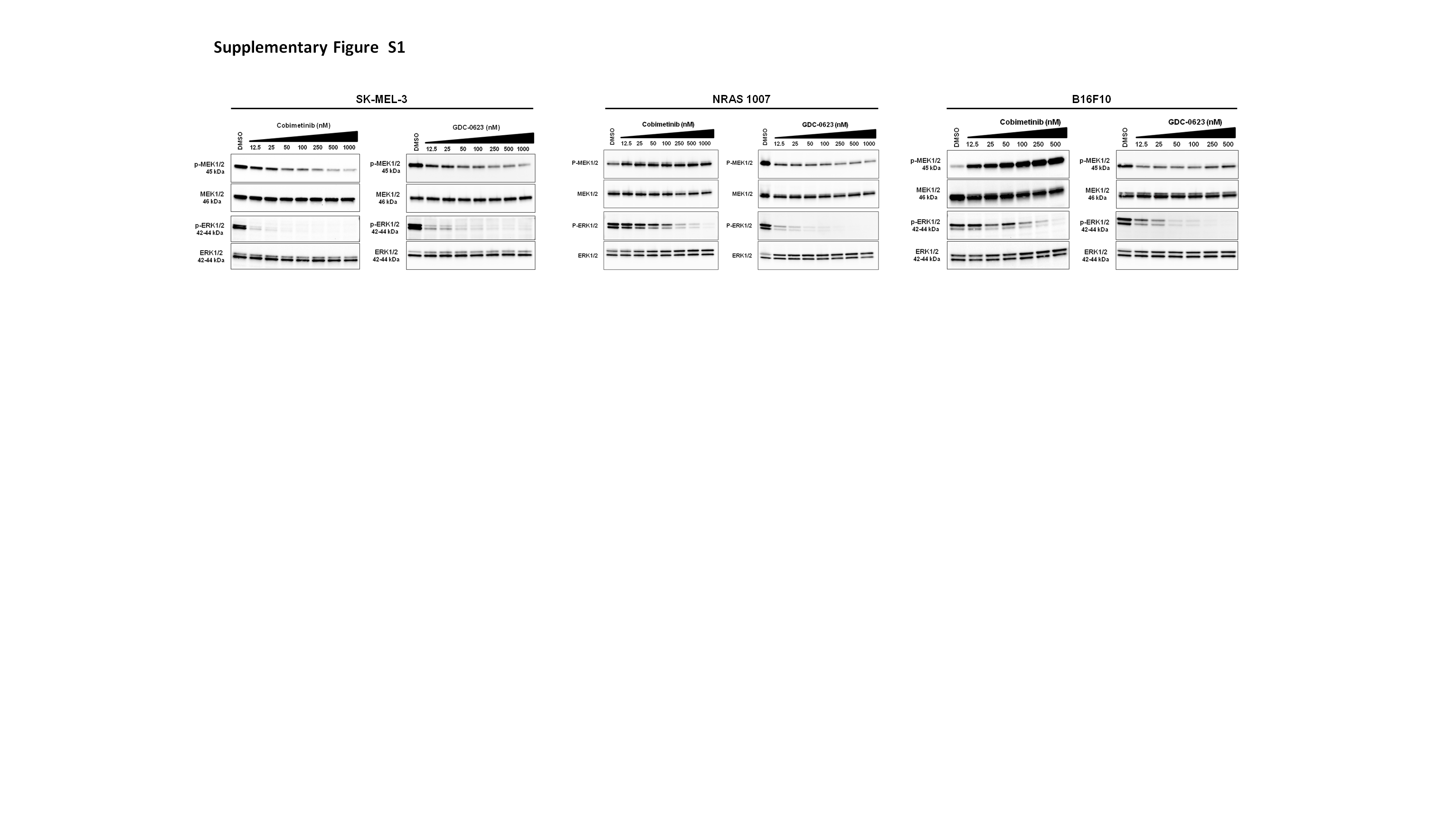

Supplement: Supplementary file 1 [file cancers-13-01421-s001.zip › Supplementary Material/Akil et al Supplementary Figure S1.TIF]

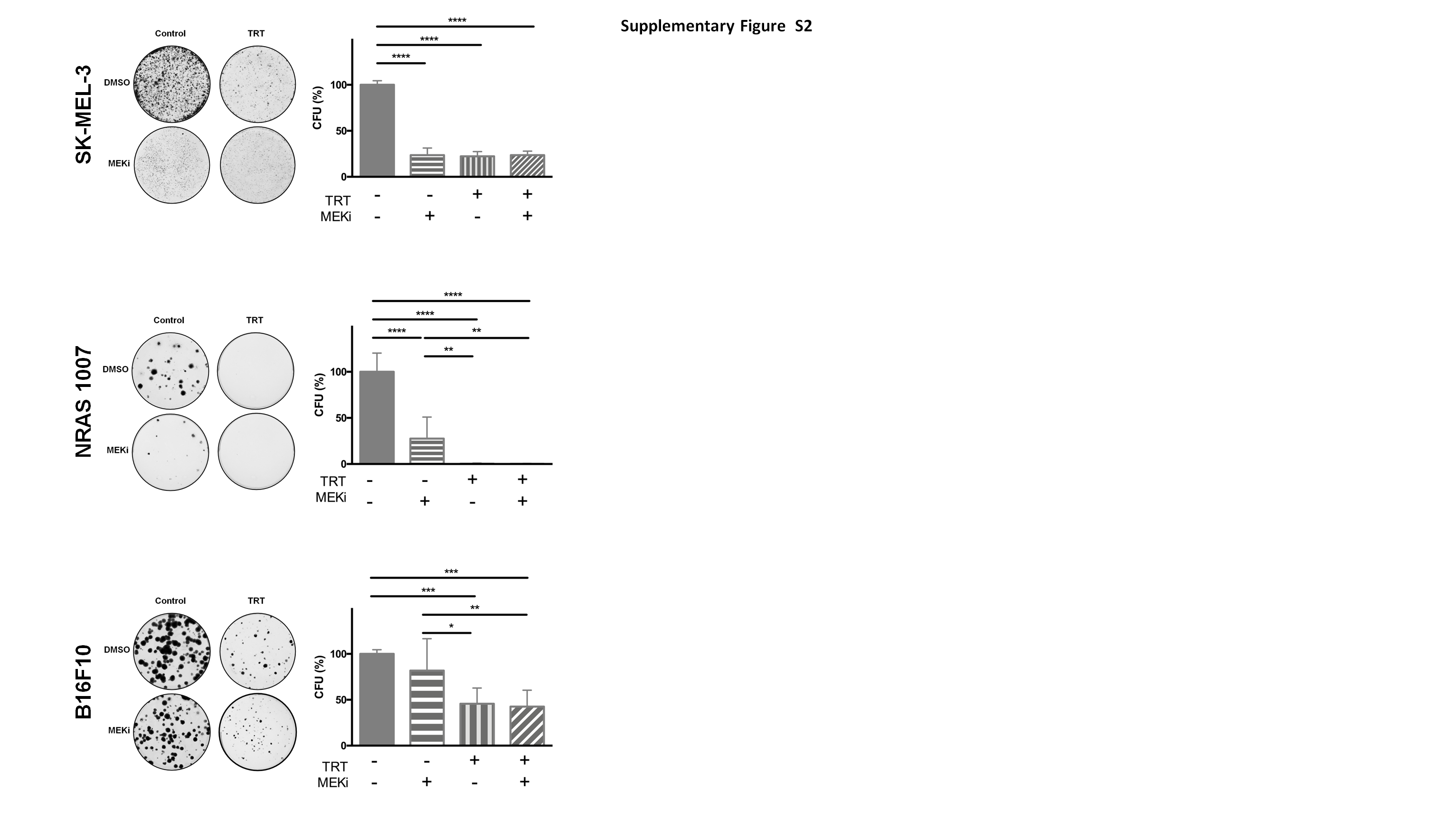

Supplement: Supplementary file 1 [file cancers-13-01421-s001.zip › Supplementary Material/Akil et al Supplementary Figure S2.TIF]

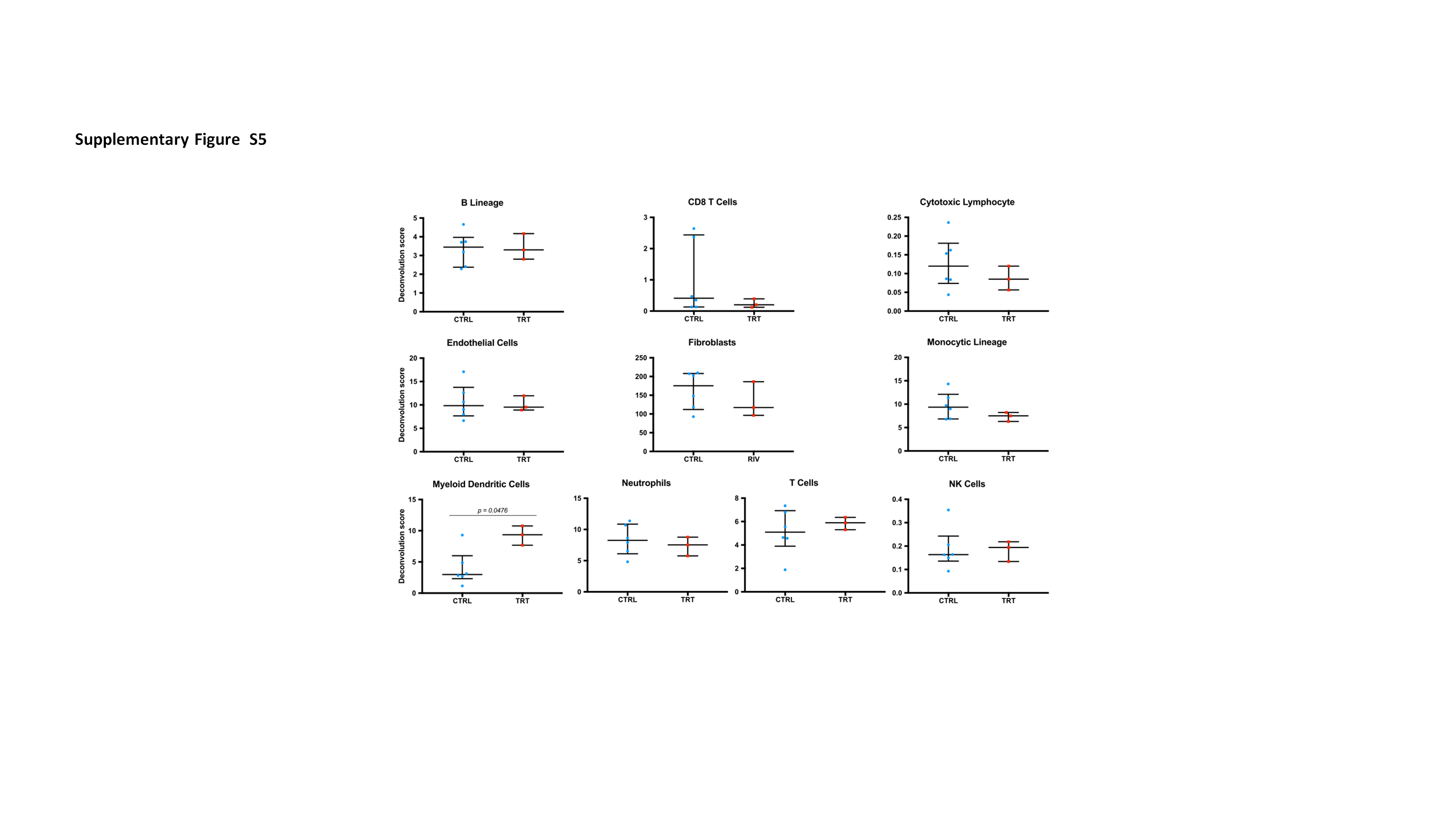

Supplement: Supplementary file 1 [file cancers-13-01421-s001.zip › Supplementary Material/Akil et al Supplementary Figure S5.TIF]

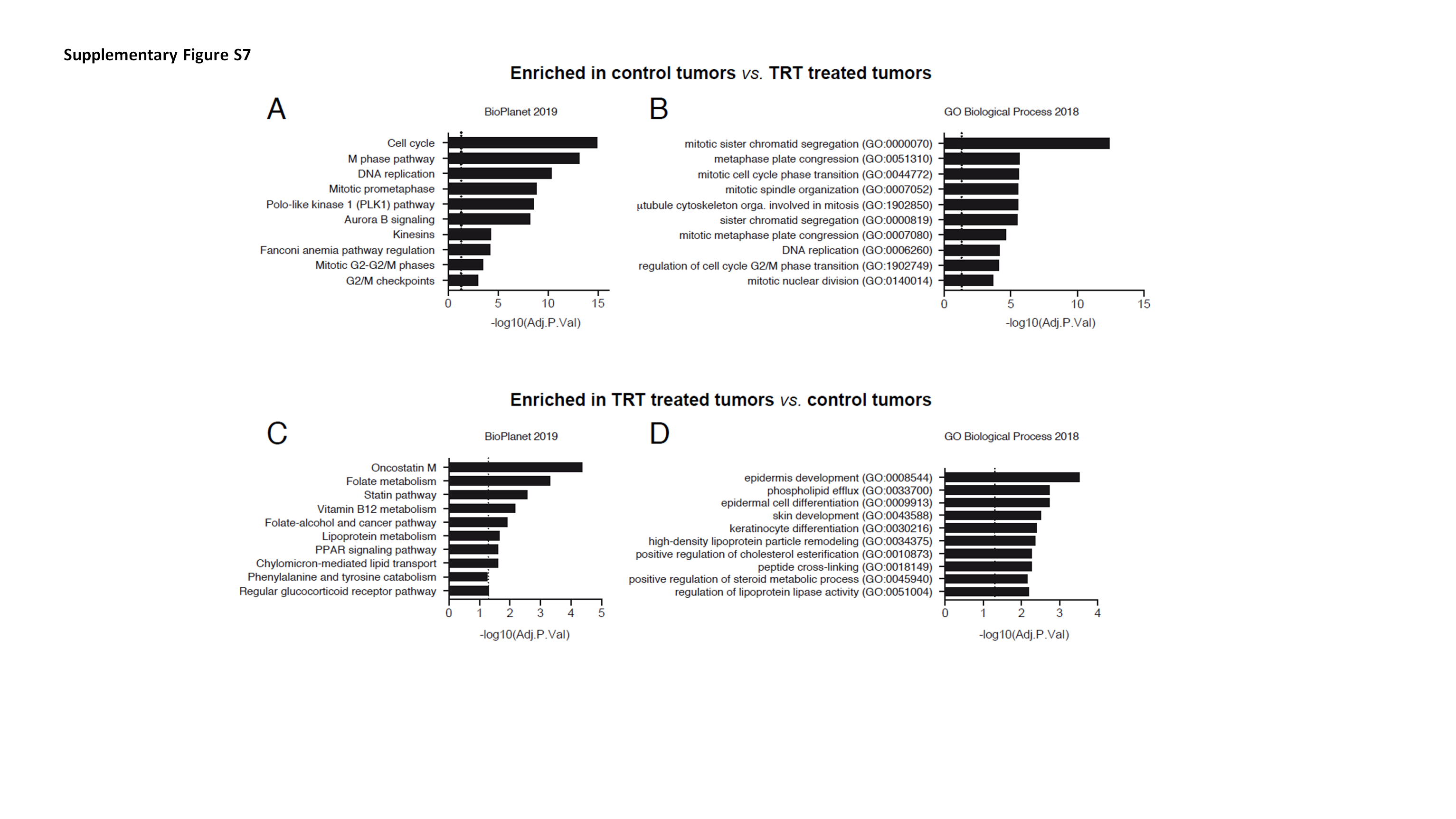

Supplement: Supplementary file 1 [file cancers-13-01421-s001.zip › Supplementary Material/Akil et al Supplementary Figure S7.TIF]

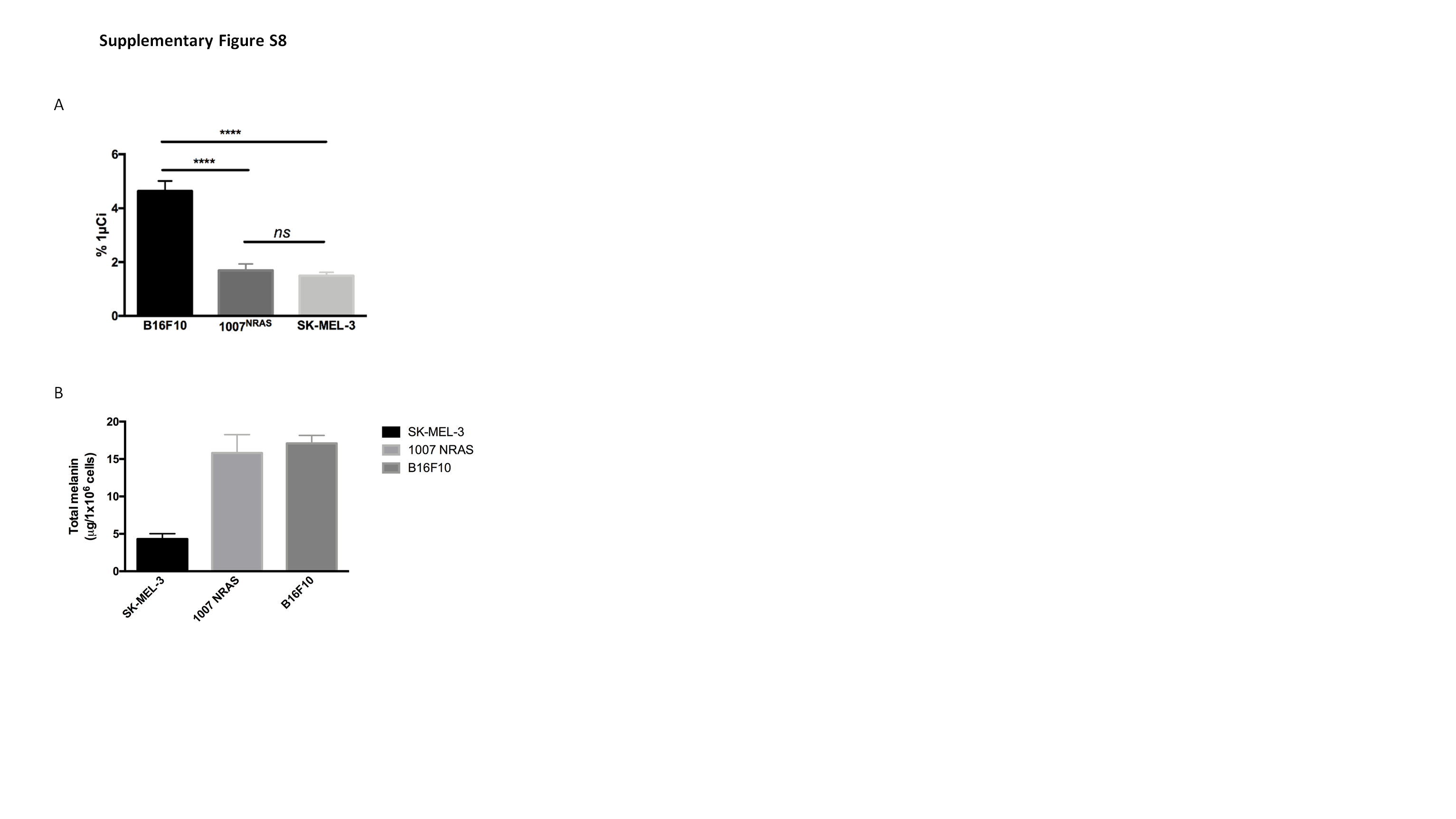

Supplement: Supplementary file 1 [file cancers-13-01421-s001.zip › Supplementary Material/Akil et al Supplementary Figure S8.TIF]

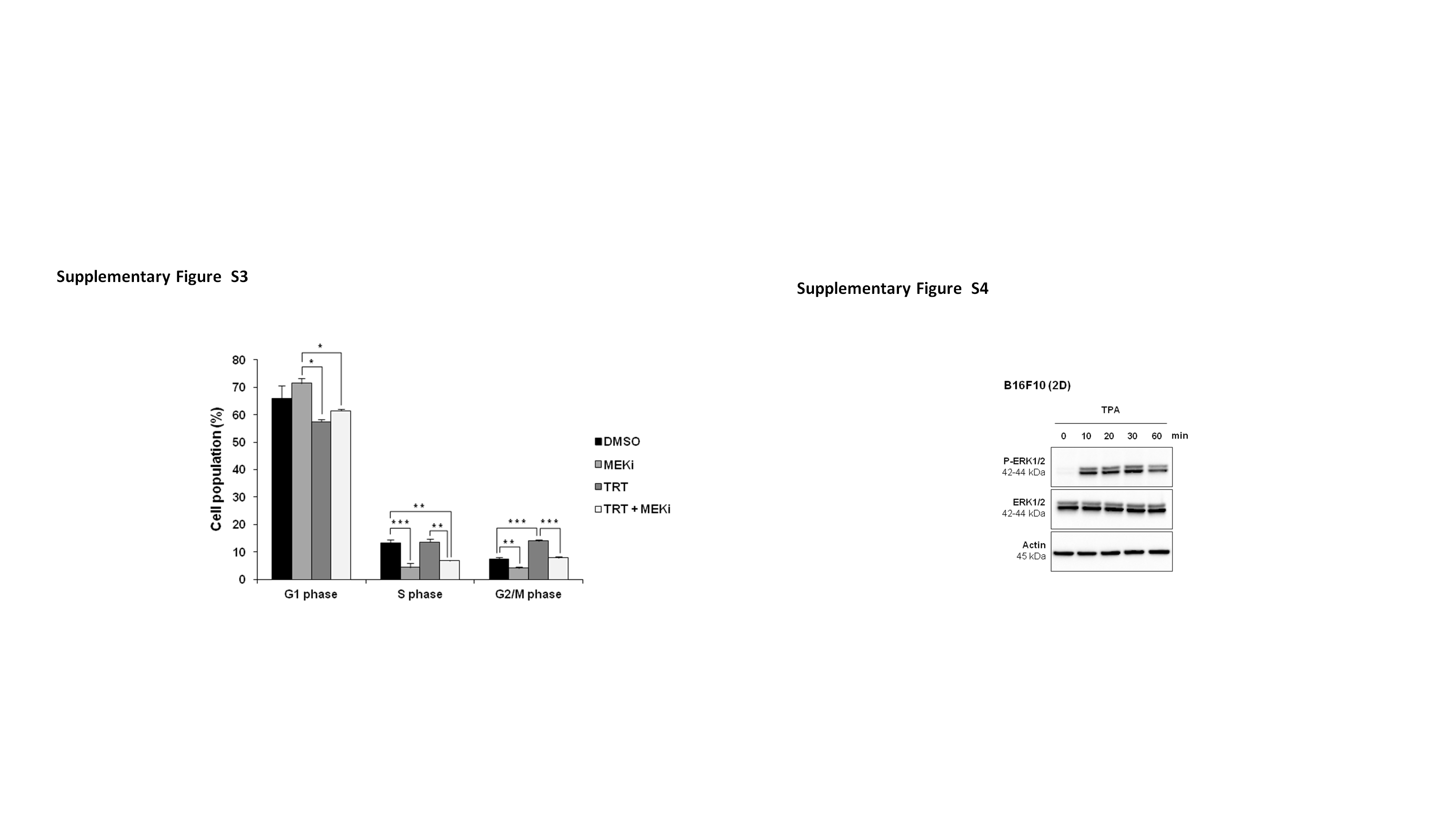

Supplement: Supplementary file 1 [file cancers-13-01421-s001.zip › Supplementary Material/Akil et al Supplementary Figures S3 & S4.TIF]
